# From spectacle to movement? Associations between major sport event perception and sport participation behavior among Host-City residents

**DOI:** 10.3389/fpubh.2026.1904469

**Published:** 2026-08-04

**Authors:** Qian Zhang, Tianyi Zhang, Zeqiang Xie

**Affiliations:** School of Social Sports, Shenyang Sport University, Shenyang, China

**Keywords:** behavioral intention, event perception, exercise, host-city residents, major sport events, public health, sport participation, theory of planned behavior

## Abstract

**Background:**

Major sport events are increasingly discussed in relation to public health because they may create a social context in which sport and physical activity become more visible to host-city residents. However, the psychological pathways through which residents’ perceptions of such events are associated with sport participation intention and behavior remain insufficiently understood.

**Methods:**

A post-event cross-sectional survey was conducted among Guangzhou residents following the 15th National Games. A structured questionnaire was used to measure event perception, attitude toward sport participation, subjective norm, perceived behavioral control, participation intention, and sport participation behavior. A total of 652 valid responses were analyzed using reliability analysis, confirmatory factor analysis, structural equation modeling, and bootstrap analysis of indirect associations.

**Results:**

Event perception was positively associated with attitude toward sport participation, subjective norm, and perceived behavioral control. Attitude, subjective norm, and perceived behavioral control were positively associated with participation intention. Participation intention and perceived behavioral control were positively associated with sport participation behavior. Bootstrap results indicated significant indirect associations between event perception and sport participation behavior through TPB constructs and participation intention.

**Conclusion:**

The findings suggest that residents’ perceptions of a major sport event may be linked to sport participation behavior through sport-related cognition, intention, and perceived behavioral control. Because the study used a cross-sectional design, the results should be interpreted as associations rather than evidence of event-generated behavioral change. These findings provide a basis for future longitudinal research examining the public-health relevance of major sport events in host-city contexts.

## 1 Introduction

Physical inactivity has become a major public health concern worldwide, contributing to increased risks of chronic diseases, reduced physical function, and lower quality of life (1). Promoting regular sport participation and physical activity is therefore an important strategy for improving population health and supporting healthier communities (2). In China, the promotion of national fitness, healthy lifestyles, and active participation in sport has become increasingly connected with broader public health goals (3). Against this background, understanding how residents develop intentions and behaviors related to leisure-time sport and exercise participation is important for designing effective community-level health promotion strategies (4).

Major sport events are increasingly discussed not only in relation to economic growth, tourism development, urban image enhancement, and infrastructure improvement, but also in relation to broader social and public-health relevance (5). In host cities, such events may increase public attention to sport, strengthen community sport atmosphere, enhance civic pride, and create opportunities for residents to become more aware of the value of physical activity (6). However, the relationship between hosting a major sport event and residents’ sport participation is not automatic. Event exposure alone is unlikely to produce lasting behavioral change (7). Therefore, it is necessary to examine whether residents’ event-related perceptions are associated with sport-related cognition, participation intention, and self-reported sport participation behavior.

Previous studies on major sport events have provided important evidence regarding economic impacts, destination image, resident support, place attachment, perceived social impacts, psychic income, event inspiration, and event benefits (8, 9). These studies have deepened understanding of how residents evaluate sport events and why they may support event hosting (10). Nevertheless, less attention has been paid to the association between residents’ event-specific perceptions and their own sport participation intention and behavior. This gap is important because residents may perceive a sport event as meaningful, exciting, or beneficial for the host city without necessarily becoming regular sport participants themselves (11).

A key conceptual issue is the transition from a spectator- or resident-oriented appraisal of the event to a participant-oriented behavioral domain. Perceiving an event positively is not the same as intending to engage in sport or reporting regular participation. For this reason, the present study does not assume that event perception directly causes sport participation. Instead, it examines whether event perception is associated with the proximal psychological constructs proposed by the Theory of Planned Behavior (TPB), namely attitude toward sport participation, subjective norm, and perceived behavioral control (12).

The TPB provides a useful framework for examining this association (13). According to the TPB, behavioral intention is the most proximal predictor of behavior, while intention is explained by attitude toward the behavior, subjective norm, and perceived behavioral control (14). This framework has been widely applied in studies of exercise, physical activity, and other health-related behaviors because it captures both motivational factors and perceived constraints (15). In the context of sport participation, residents are more likely to report intention to participate when they evaluate sport participation positively, perceive support from significant others and the broader community, and believe that they have sufficient time, resources, opportunities, and ability to participate. Perceived behavioral control may also be directly associated with sport participation behavior because actual participation depends not only on intention but also on perceived feasibility and control over practical conditions (16).

Although the TPB has strong explanatory power, it mainly focuses on proximal psychological determinants and pays relatively limited attention to the contextual conditions under which these determinants are formed (17). In host-city settings, major sport events may serve as an important contextual factor. Residents’ perceptions of the 15th National Games may be associated with sport-related attitudes by increasing the perceived value and attractiveness of sport participation. Event perception may also be associated with subjective norms by increasing the visibility of sport in public discussion, family interaction, community life, and social networks (18). In addition, positive event perception may be linked to perceived behavioral control if residents become more aware of sport atmosphere, access to facilities, public sport services, or opportunities for participation (19).

The 15th National Games in Guangzhou provides an appropriate context for examining this issue. As a major national sport event embedded in the host-city environment, the Games created a public context in which sport, urban identity, community engagement, and health-oriented lifestyles became more salient. For Guangzhou residents, perceptions of the event may not only reflect support for the Games, but also be associated with how they evaluate sport participation, perceive social support, and assess their ability to engage in regular leisure-time sport and exercise participation.

Accordingly, this study aims to examine the associations between event perception and sport participation behavior among host-city residents in Guangzhou. Specifically, this study develops and tests an extended TPB model in which event perception is treated as a contextual antecedent associated with attitude toward sport participation, subjective norm, and perceived behavioral control (20). The study further examines how these TPB constructs are associated with participation intention and how participation intention and perceived behavioral control are associated with sport participation behavior (21). By doing so, this study contributes to sport-event research by clarifying a potential association-based pathway from event-related appraisal to sport participation outcomes, while avoiding causal claims that cannot be supported by cross-sectional data (22).

## 2 Theoretical framework and hypotheses

### 2.1 Extended theory of planned behavior

The Theory of Planned Behavior (TPB) provides the theoretical foundation for this study. The TPB proposes that individual behavior is primarily determined by behavioral intention, while intention is shaped by three social-cognitive factors: attitude toward the behavior, subjective norm, and perceived behavioral control (13). Attitude refers to an individual’s favorable or unfavorable evaluation of performing a behavior. Subjective norm reflects perceived social expectations, support, or pressure from significant others. Perceived behavioral control refers to the perceived ease or difficulty of performing a behavior, based on available resources, opportunities, abilities, and confidence (16).

The TPB is particularly relevant for explaining sport participation and exercise-related behavior (23). In this study, sport participation refers to residents’ voluntary engagement in structured or semi-structured sport and exercise activities during leisure time (24). It is treated as a specific form of health-related physical activity rather than as a broad measure of total physical activity across occupational, transport, household, and leisure domains. Regular sport participation is not determined only by awareness of health benefits. It also depends on whether individuals evaluate sport participation positively, perceive support from family members, friends, peers, and the broader community, and believe that they have sufficient time, resources, opportunities, and ability to participate.

Although the TPB is a robust framework for explaining proximal psychological determinants of behavior, it pays relatively limited attention to the contextual conditions under which attitudes, norms, and perceived control are formed (25). In host-city settings, major sport events may function as salient contextual stimuli. Through event communication, public sport atmosphere, community activities, media exposure, and civic pride, major sport events may be associated with residents’ sport-related cognition and motivation. Therefore, this study extends the TPB by incorporating event perception as a contextual antecedent of attitude toward sport participation, subjective norm, and perceived behavioral control. This extended model allows us to examine whether residents’ perception of the 15th National Games is associated with sport participation behavior through both contextual appraisal and individual psychological constructs.

### 2.2 Event perception and TPB constructs

In this study, event perception is conceptualized as host-city residents’ event-specific contextual appraisal of the 15th National Games as a sport-related public environment. It refers to the extent to which residents perceive the event as making sport more visible, socially salient, symbolically meaningful, and relevant to everyday sport participation in Guangzhou (26). Therefore, event perception does not simply denote exposure to the event, general support for hosting, or a broad evaluation of all event outcomes. Rather, it captures residents’ appraisal of the event context that may be associated with sport-related attitudes, perceived social expectations, and perceived opportunities for participation (27).

This conceptualization requires clarification because several related constructs have been widely discussed in the sport-event legacy literature. Event perception differs from event inspiration, which usually refers to an affective-motivational state through which an event stimulates individuals to try, resume, or increase sport participation (28). By contrast, event perception in this study is not defined as an immediate motivational impulse, but as residents’ appraisal of the event as part of the local sport and community environment.

Event perception also differs from psychic income. Psychic income mainly captures intangible psychological benefits associated with event hosting, such as pride, excitement, happiness, and a feel-good effect (29). Although the present construct includes affective elements such as host-city pride and community excitement, its theoretical role is not to measure emotional benefits as legacy outcomes. Instead, it is used to examine whether residents’ event-related appraisals are associated with the proximal social-cognitive determinants of sport participation specified by the TPB.

Similarly, event perception should not be equated with perceived social impact. Perceived social impact usually refers to residents’ assessment of broader positive and negative social consequences of event hosting, including social cohesion, community development, cultural exchange, crowding, disruption, and quality-of-life effects (30). The present study adopts a narrower behavioral focus. It examines only those event-related appraisals that are theoretically relevant to sport participation, such as the perceived strengthening of the local sport atmosphere, the visibility of sport in community life, and the perceived cultural and public value of sport.

Event perception is also distinct from event involvement and place attachment. Event involvement concerns the degree of residents’ personal interest, attention, or engagement with the event itself, such as watching competitions, following event news, volunteering, or participating in event-related activities. Event perception, however, may be formed even among residents who are not highly involved in the event, because it concerns their appraisal of the event as part of the broader host-city environment. Place attachment refers to a relatively stable emotional and cognitive bond between individuals and a place. Although the Games may activate a sense of belonging or host-city pride, the construct examined here is not residents’ general attachment to Guangzhou. It refers specifically to event-activated host-city meaning in the context of the 15th National Games.

A key theoretical issue in linking event perception to sport participation is the transition from a spectator- or resident-oriented appraisal of the event to a participant-oriented behavioral domain. This study does not assume that event perception directly produces sport participation. Rather, it treats event perception as a contextual appraisal that may make sport more salient, socially visible, symbolically meaningful, and practically relevant in residents’ everyday lives. Major sport events can expose residents to sport-related narratives, community activities, media communication, public discussion, and visible improvements in the local sport environment (31). Through these processes, residents may evaluate sport participation more favorably, perceive stronger social support for participation, and become more aware of opportunities and resources that make participation feasible.

The distinctiveness of event perception also lies in its object and theoretical function. Its object is the major sport event as a contextual stimulus, whereas the object of attitude in the TPB is the behavior of regular sport participation (12). A resident may evaluate the 15th National Games positively as a city event but still consider regular sport participation difficult, inconvenient, or unenjoyable. Conversely, a resident may hold a favorable attitude toward sport participation without perceiving the Games as especially meaningful. For this reason, event perception and attitude toward sport participation are theoretically distinguishable even though both contain evaluative elements.

Event perception may be associated with residents’ attitude toward sport participation. According to the TPB, attitude is formed through beliefs about the expected outcomes of performing a behavior and the value attached to those outcomes. When residents perceive that the 15th National Games strengthens the sport atmosphere of the city, increases the visibility of sport in daily life, and enhances the symbolic value of sport participation, they may be more likely to view regular sport participation as beneficial, enjoyable, meaningful, and worthwhile (32). Therefore, the following hypothesis is proposed:

*H1*: Event perception is positively associated with attitude toward sport participation.

Event perception may also be associated with subjective norm regarding sport participation. Major sport events are usually accompanied by media coverage, public discussion, community mobilization, and collective attention (33). These processes may increase the social visibility and perceived legitimacy of sport participation. When sport becomes more visible in the host-city environment, residents may perceive stronger support or approval from family members, friends, colleagues, peers, and the broader community (34). Therefore, the following hypothesis is proposed:

*H2*: Event perception is positively associated with subjective norm regarding sport participation.

Event perception may further be associated with perceived behavioral control. Perceived behavioral control reflects whether individuals believe that they have the resources, opportunities, ability, and confidence required to perform a behavior. In the context of a major sport event, residents who perceive the event positively may become more aware of sport facilities, public sport programs, participation opportunities, and community sport resources. They may also perceive the urban sport environment as more supportive and accessible (35). Therefore, the following hypothesis is proposed:

*H3*: Event perception is positively associated with perceived behavioral control regarding sport participation.

### 2.3 Attitude toward sport participation and participation intention

Attitude toward sport participation refers to residents’ overall evaluation of engaging in regular sport or exercise activities (36). In the TPB, attitude is regarded as a central determinant of behavioral intention because individuals are more likely to intend to perform a behavior when they evaluate it positively (21, 32). In the public health context, residents may develop favorable attitudes toward sport participation when they believe that regular physical activity can improve health, enhance physical function, reduce stress, provide enjoyment, increase social interaction, and improve quality of life.

Sport participation is a voluntary behavior that requires time allocation, effort, and self-regulation. Residents who believe that sport participation is beneficial and meaningful are more likely to report stronger intention to participate (37). By contrast, if residents view sport participation as inconvenient, uninteresting, or unrelated to their daily needs, they may be less likely to develop such intention. Therefore, attitude toward sport participation is expected to be associated with participation intention. Accordingly, the following hypothesis is proposed:

*H4*: Attitude toward sport participation is positively associated with participation intention.

### 2.4 Subjective norm and participation intention

Subjective norm refers to perceived social expectations, approval, or support regarding whether an individual should perform a behavior. In the context of sport participation, subjective norm captures residents’ perceptions that important others, such as family members, friends, colleagues, peers, and community members, support or encourage their participation in sport (34). Sport participation is often embedded in interpersonal relationships and community life (38). Therefore, social support and perceived approval may be associated with residents’ willingness to engage in regular sport activities.

In host-city contexts, the social influence mechanism may become more salient because major sport events can increase public discussion and collective attention to sport. When residents perceive that people around them value sport participation and regard it as a desirable and healthy lifestyle, they may be more likely to form an intention to participate (31). Although subjective norm may not always be the strongest predictor of intention in physical activity research, it remains an important source of social motivation, especially in community-based sport participation. Therefore, the following hypothesis is proposed:

*H5*: Subjective norm is positively associated with participation intention.

### 2.5 Perceived behavioral control, participation intention, and sport participation behavior

Perceived behavioral control refers to residents’ perceived capacity to participate in sport, including their perceived time, resources, opportunities, physical ability, and confidence. This construct is important because regular sport participation is not entirely under volitional control. Even when residents have positive attitudes and strong intentions, actual participation may still be constrained by work responsibilities, family obligations, lack of facilities, limited time, health conditions, or insufficient access to suitable activities (37).

Perceived behavioral control may be associated with participation intention. When residents believe that they have sufficient ability, time, and opportunity to participate in sport, they are more likely to report stronger intention to engage in regular sport activities (39). Conversely, when they perceive sport participation as difficult or constrained by external conditions, their intention may be weakened. Therefore, the following hypothesis is proposed:

*H6*: Perceived behavioral control is positively associated with participation intention.

### 2.6 Participation intention and sport participation behavior

Participation intention refers to residents’ motivational readiness and deliberate plan to engage in regular sport activities (40). In the TPB, intention is theoretically positioned as a proximal determinant of behavior because it reflects the degree of willingness, planning, and effort that individuals are prepared to invest in performing a behavior. In the present cross-sectional study, however, the empirical relationship between participation intention and sport participation behavior is examined as a theory-specified association rather than as prospective behavioral prediction.

Regular sport participation requires sustained effort rather than a one-time decision (41). Residents who report stronger participation intention are expected to report higher levels of sport participation in daily life (42). However, the intention-behavior gap should also be acknowledged. Some residents may intend to participate but fail to translate intention into behavior due to practical constraints (43, 44). For this reason, this study examines the associations of participation intention and perceived behavioral control with sport participation behavior. Based on the TPB, the following hypotheses are proposed:

*H7*: Participation intention is positively associated with sport participation behavior.

*H8*: Perceived behavioral control is positively associated with sport participation behavior.

### 2.7 Hypothesized model

Based on the above theoretical reasoning, this study develops an extended TPB model to examine sport participation behavior among host-city residents of the 15th National Games in Guangzhou. In this model, event perception is treated as a contextual antecedent associated with attitude toward sport participation, subjective norm, and perceived behavioral control. These three TPB constructs are expected to be associated with participation intention, while participation intention and perceived behavioral control are expected to be associated with sport participation behavior.

In addition to the direct associations specified above, this study further examines indirect associations between event perception and sport participation behavior through TPB constructs and participation intention (45). Because the cross-sectional design does not allow causal mediation to be established, these hypotheses are framed as indirect associations rather than confirmed temporal mechanisms.

*H9a*: Event perception is indirectly associated with sport participation behavior through attitude toward sport participation and participation intention.

*H9b*: Event perception is indirectly associated with sport participation behavior through subjective norm and participation intention.

*H9c*: Event perception is indirectly associated with sport participation behavior through perceived behavioral control and participation intention.

**Figure 1:**
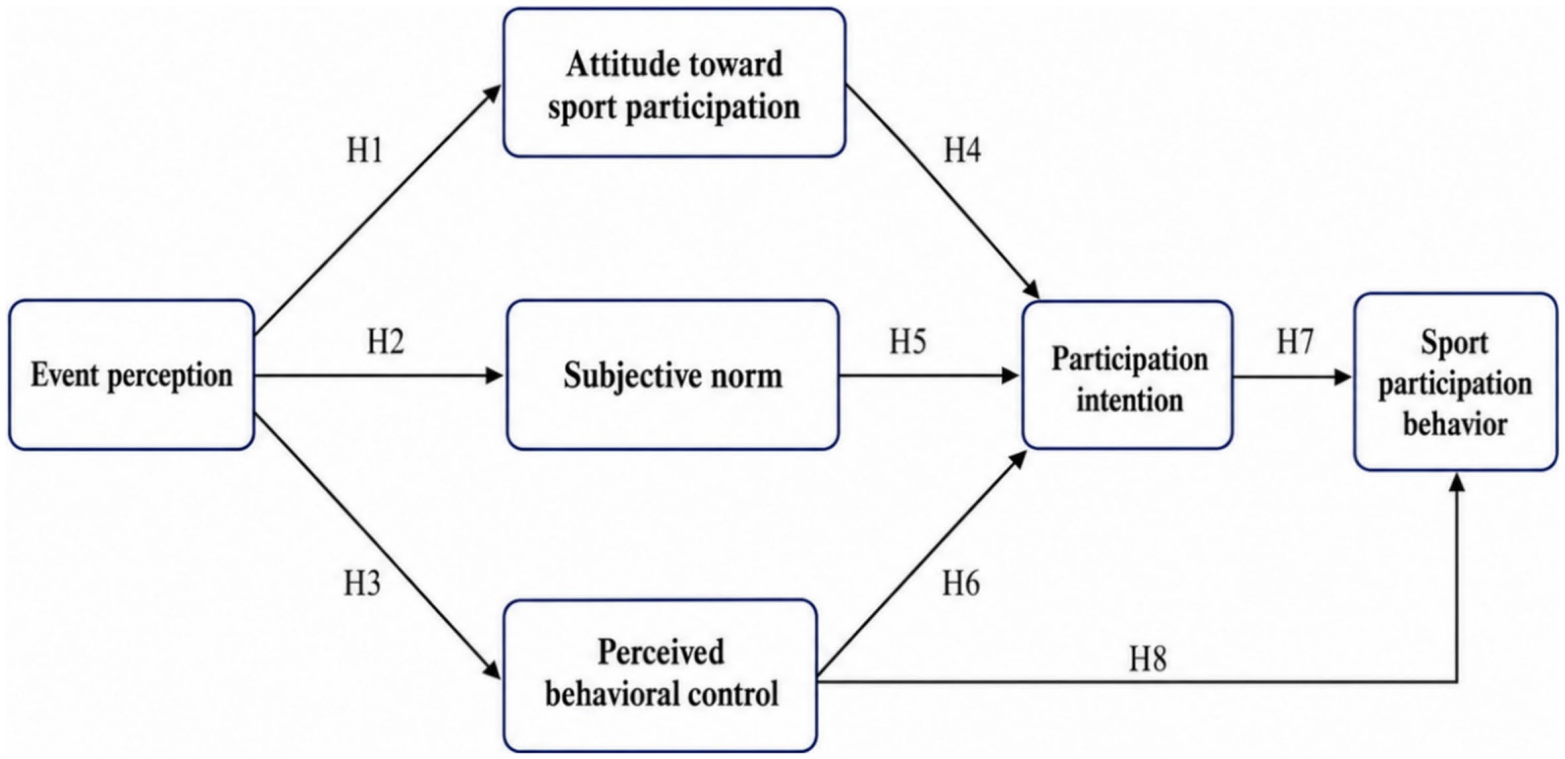
Hypothesized model.

The hypothesized model is shown in Figure 1.

## 3 Methods

### 3.1 Study design and setting

This study adopted a cross-sectional questionnaire survey design to examine the association between event perception and sport participation behavior among host-city residents. The study was conducted in Guangzhou, China, in the context of the 15th National Games. Guangzhou was selected as the research setting because the event provided a meaningful host-city context in which sport, urban identity, community engagement, and physical activity promotion became more visible in residents’ daily lives (46).

The target population consisted of adult residents living in Guangzhou. A structured questionnaire was used to collect data on residents’ event perception, attitude toward sport participation, subjective norm, perceived behavioral control, participation intention, and sport participation behavior (47). The overall study design was intended to test an extended TPB model and to clarify whether event perception was associated with residents’ sport participation behavior through TPB constructs. Because the design was cross-sectional, the study was not intended to establish causal effects or event-generated behavioral change.

### 3.2 Participants and sampling

Participants were eligible for inclusion if they met the following criteria: (a) aged 18 years or older; (b) had continuously resided in Guangzhou for at least 6 months; (c) were able to understand and complete the questionnaire independently; and (d) voluntarily agreed to participate in the study. Respondents who did not meet the residence criterion, provided incomplete responses, or submitted questionnaires with obvious response patterns or logical inconsistencies were excluded (48).

Because a complete sampling frame of Guangzhou residents was not available, this study used a non-probability sampling strategy combining convenience sampling and snowball sampling (49, 50). This approach was considered appropriate for reaching residents from different communities and administrative districts in the host city. To increase sample diversity, the questionnaire was distributed through both online and offline channels (51). A total of 759 questionnaires were collected. After data screening, 652 valid responses were retained for analysis, representing a retention rate of 85.9% after data screening.

### 3.3 Data collection procedure

Data were collected from December 2025 to March 2026, following the conclusion of the 15th National Games. The formal data collection reported in this section refers only to the main survey. The 35 Guangzhou residents who participated in the pilot test were not included in the final dataset used for analysis. The questionnaire was distributed through online and offline channels (51). Online questionnaires were disseminated through an electronic survey platform, social media, community groups, and respondent referrals. Offline questionnaires were distributed in community spaces, public activity areas, university surroundings, and sport-related settings in Guangzhou.

Before completing the questionnaire, participants were informed of the purpose of the study, the voluntary nature of participation, the anonymity of responses, and the academic use of the data. No personally identifiable information was collected. Participants were also informed that they could stop answering the questionnaire at any time before submission. To ensure data quality, questionnaires with excessive missing values, unusually short completion times, patterned responses, inconsistent answers, or failure to meet the inclusion criteria were removed before Formal analysis (48).

### 3.4 Measurement instruments

A structured questionnaire was developed through a literature-based adaptation and contextualization process (52). The questionnaire was not developed *de novo*; rather, it was adapted from established theoretical constructs and previous empirical studies related to the Theory of Planned Behavior, sport-event legacy, psychic income, perceived social impact, event inspiration, event leveraging, and sport and exercise participation. Based on this literature, the initial questionnaire was contextualized for the 15th National Games in Guangzhou. The questionnaire consisted of three sections: study information and consent, demographic information, and measures of event perception, attitude toward sport participation, subjective norm, perceived behavioral control, participation intention, and sport participation behavior. The constructs, measurement items, and corresponding sources are presented in Table 1.

**Table 1:** Constructs, measurement items, and sources.

Construct	Item	Measurement item	Source
Event perception	EP1	The 15th National Games made sport more visible in Guangzhou’s everyday urban life.	(27, 53)
	EP2	The 15th National Games created a clear festive atmosphere and collective excitement around sport.	
	EP3	The 15th National Games made me feel more connected to Guangzhou as a host city.	
	EP4	As a resident of Guangzhou, I felt proud that the 15th National Games was hosted locally.	
	EP5	The 15th National Games improved my positive evaluation of Guangzhou’s city image in the event context.	
	EP6	The 15th National Games enhanced my understanding of sport culture and the public value of sport events.	
Attitude toward sport participation	ATT1	For me, maintaining regular sport participation in the next month would be beneficial.	(47, 54)
	ATT2	For me, maintaining regular sport participation in the next month would be worthwhile.	
	ATT3	For me, maintaining regular sport participation in the next month would be pleasant.	
	ATT4	For me, maintaining regular sport participation in the next month would be meaningful.	
	ATT5	For me, maintaining regular sport participation in the next month would be enjoyable.	
Subjective norm	SN1	Most people who are important to me support my regular participation in sport in the next month.	(13, 47)
	SN2	My family members or friends would approve of my engaging in sport at least three times per week, for at least 30 min each time, in the next month.	
	SN3	People like me generally maintain regular sport participation in their daily lives.	
Perceived behavioral control	PBC1	I am confident that I can maintain regular sport participation in the next month.	(16, 47)
	PBC2	Even if I am busy with study, work, or family responsibilities, I can arrange time for sport participation.	
	PBC3	Whether I maintain regular sport participation in the next month mainly depends on myself.	
	PBC4	For me, engaging in sport at least three times per week, for at least 30 min each time, in the next month would be easy to do.	
Participation intention	INT1	I intend to engage in sport at least three times per week, for at least 30 min each time, in the next 4 weeks.	(40, 47)
	INT2	I plan to maintain regular sport participation in the next 4 weeks.	
	INT3	I have a clear intention to maintain regular sport participation in the next 4 weeks.	
	INT4	I will make an effort to engage in sport at least three times per week, for at least 30 min each time, in the next 4 weeks.	
Sport participation behavior	BEH1	In the past 4 weeks, how many times per week did you participate in sport on average?	(24, 55)
	BEH2	In the past 4 weeks, what was the average duration of each sport participation session?	
	BEH3	In the past 4 weeks, what was the usual intensity of your sport participation?	
	BEH4	In the past 4 weeks, how many weeks did you meet the standard of “at least three times per week, with each session lasting at least 30 min”?	

Except for sport participation behavior, all constructs were measured using five-point Likert-type items. Sport participation behavior was measured using 4 five-level ordered items referring to leisure-time sport/exercise participation during the past 4 weeks. The event perception items refer to appraisal of the 15th National Games as an event context, whereas attitude items refer to evaluation of regular sport participation as a personal behavior.

Before the formal survey, the initial questionnaire was reviewed by three researchers with expertise in sport management, physical activity research, and survey methodology (52). The review focused on construct relevance, wording clarity, contextual appropriateness, and consistency between the theoretical constructs and their measurement items. Particular attention was paid to the distinction between event perception and attitude toward sport participation. Specifically, the event perception items were examined to ensure that they referred to residents’ appraisal of the 15th National Games as an event context, whereas the attitude items referred to residents’ evaluation of regular sport participation as a personal behavior.

After the expert review, a pilot test was conducted with 35 Guangzhou residents who met the inclusion criteria. These pilot participants were not included in the formal survey sample, the 759 collected questionnaires, or the final analytic sample of 652 respondents. The pilot test was used to assess item comprehensibility, completion time, response-category clarity, and contextual suitability. Based on the expert review and pilot feedback, only minor wording revisions were made. These revisions mainly involved clarifying references to the 15th National Games, simplifying expressions that might be difficult for respondents to understand, distinguishing the object of event perception from the object of sport participation attitude, and ensuring consistency in the use of the terms sport participation, exercise, and physical activity. No core construct was removed after the pilot test. The pilot test was used to improve item clarity and contextual appropriateness rather than to conduct a separate scale validation. The reliability and validity of the measurement model were formally assessed using the final analytic sample of 652 valid responses.

Event perception was measured using six items assessing residents’ event-specific appraisal of the 15th National Games, including the perceived strengthening of Guangzhou’s sport atmosphere, collective excitement, host-city pride activated by the Games, event-related sense of belonging, positive evaluation of the city image in the event context, and enhanced understanding of sport culture and the public value of sport events (27, 53). These items were intended to measure residents’ appraisal of the event as a contextual stimulus. Attitude toward sport participation, by contrast, was measured using five items assessing residents’ evaluation of maintaining regular sport participation as a personal behavior (47, 54).

Subjective norm was measured using three items adapted from TPB questionnaire guidance (13, 47), capturing perceived approval and support from important others as well as perceived participation patterns among similar others. Perceived behavioral control was measured using four items adapted from TPB-based measures of perceived capacity and controllability (16, 47). The items assessed confidence, perceived time availability, controllability, and the perceived ease of maintaining regular sport participation. Participation intention was measured using four items assessing residents’ willingness, plans, and effort to participate in regular sport activities in the near future (40, 47). Except for sport participation behavior, all psychological constructs were measured using five-point Likert-type items ranging from 1 = strongly disagree to 5 = strongly agree.

In this study, sport participation refers to residents’ voluntary engagement in structured or semi-structured sport and exercise activities during leisure time. It is treated as a specific form of health-related physical activity rather than as a broad measure of total physical activity across occupational, transport, household, and leisure domains. Sport participation behavior was operationalized as leisure-time sport and exercise participation during the past 4 weeks and measured using four five-level ordered behavioral response items: exercise frequency, average duration per session, usual exercise intensity, and regularity of meeting the criterion of at least three times per week, with each session lasting at least 30 min (24, 55). For the SEM analysis, the four behavioral indicators were modeled as reflective indicators of a latent sport participation behavior construct representing a common underlying level of leisure-time sport participation. Responses were coded from 1 to 5, with higher values indicating higher levels of sport participation behavior. The full measurement items and response categories are provided in Supplementary Table S1.

### 3.5 Ethical considerations

Participation in this study was voluntary and anonymous. All respondents were informed of the research purpose, the academic use of the data, and their right to withdraw from the survey at any time before submission. No personally identifiable information was collected, and all data were used only for academic research. The questionnaire was completed only after respondents indicated their willingness to participate. The study involved an anonymous questionnaire survey with minimal risk to participants. Written or electronic informed consent was obtained from all participants before the survey.

### 3.6 Data analysis

Data analysis was conducted using IBM SPSS Statistics 26.0 and IBM AMOS 24.0 (56, 57). Before Formal analysis, the dataset was screened to identify incomplete responses, patterned answers, unusually short completion times, logical inconsistencies, and cases that did not meet the inclusion criteria (48). Descriptive statistics were then used to summarize respondents’ demographic characteristics and the distribution of the main study variables.

Common method variance was considered because all core variables were measured using self-reported questionnaire data at a single point in time (58). Procedural remedies were adopted during questionnaire design and administration, and Harman’s single-factor test was used as a preliminary statistical diagnostic (59). Reliability was evaluated using Cronbach’s alpha coefficients and corrected item-total correlations (60).

Confirmatory factor analysis was then performed to evaluate the measurement model (61). Model fit was assessed using commonly recommended fit indices, including the chi-square to degrees of freedom ratio (χ^2^/df), root mean square error of approximation (RMSEA), standardized root mean square residual (SRMR), comparative fit index (CFI), incremental fit index (IFI), and Tucker-Lewis index (TLI) (62). Convergent validity was assessed using standardized factor loadings, composite reliability, and average variance extracted (63). Discriminant validity was examined using the Fornell-Larcker criterion (64). To provide additional evidence of construct distinctiveness, the heterotrait–monotrait ratio of correlations (HTMT) was also calculated for the six constructs included in the measurement model. HTMT values were evaluated using the conservative threshold of 0.85.

The primary SEM was theory-specified and did not include demographic covariates. To assess the robustness of the focal associations, five additional hierarchical multiple linear regression models were estimated using the construct scores. Gender, age, educational attainment, monthly income, residence duration, and residential district were entered as demographic covariates in the first block, followed by the focal theoretical predictor or predictors in the second block. Categorical demographic variables were dummy coded. The adjusted analyses were used to examine whether the direction and statistical significance of the eight focal associations remained substantively consistent after demographic adjustment.

Pearson correlation analysis was used to examine the bivariate relationships among event perception, attitude toward sport participation, subjective norm, perceived behavioral control, participation intention, and sport participation behavior. Structural equation modeling was subsequently performed to test the hypothesized associations among the constructs (65). In addition, the bootstrap procedure with 5,000 resamples was used to examine indirect associations. Indirect associations were considered statistically significant when the 95% bias-corrected confidence interval did not include zero. Because the study was cross-sectional, these indirect associations were not interpreted as evidence of causal mediation.

## 4 Results

### 4.1 Participant characteristics

A total of 759 questionnaires were collected. After excluding invalid responses, 652 valid questionnaires were retained for analysis, representing a retention rate of 85.9% after data screening. The demographic characteristics of the respondents are presented in Table 2.

**Table 2:** Demographic characteristics of respondents (*N* = 652).

Variable	Category	n	%
Gender	Male	366	56.1
	Female	286	43.9
Age	18–29 years	179	27.5
	30–39 years	226	34.7
	40–49 years	146	22.4
	50–59 years	79	12.1
	60 years and above	22	3.4
Education level	Junior high school and below	52	8.0
	High school/Technical secondary school	102	15.6
	Junior college	168	25.8
	Bachelor’s degree	259	39.7
	Postgraduate degree and above	71	10.9
Occupation	Government/Institution staff	85	13.0
	Enterprise staff	240	36.8
	Self-employed/Freelancer	92	14.1
	Worker/Service industry practitioner	92	14.1
	Student	97	14.9
	Retired personnel	17	2.6
	Unstable occupation/Unemployed	20	3.1
	Other	9	1.4
Monthly income	≤ 3,000 yuan	116	17.8
	3,001–5,000 yuan	89	13.7
	5,001–8,000 yuan	194	29.8
	8,001–12,000 yuan	182	27.9
	≥ 12,001 yuan	71	10.9
Residence duration in Guangzhou	6 months–1 year	77	11.8
	1–3 years	123	18.9
	3–5 years	196	30.1
	5–10 years	161	24.7
	More than 10 years	95	14.6
Residential district in Guangzhou	Yuexiu District	66	10.1
	Haizhu District	84	12.9
	Liwan District	70	10.7
	Tianhe District	68	10.4
	Baiyun District	76	11.7
	Huangpu District	58	8.9
	Panyu District	59	9.0
	Huadu District	53	8.1
	Nansha District	47	7.2
	Conghua District	36	5.5
	Zengcheng District	35	5.4

The sample covered all 11 administrative districts of Guangzhou, indicating broad geographic coverage within the host city. Among the respondents, 366 were male (56.1%) and 286 were female (43.9%). In terms of age, most participants were young and middle-aged adults. Respondents aged 18–29 years accounted for 27.5%, those aged 30–39 years accounted for 34.7%, and those aged 40–49 years accounted for 22.4%. Overall, respondents aged 18–49 years represented 84.5% of the total sample.

Regarding educational attainment, respondents with a bachelor’s degree formed the largest group (39.7%), followed by those with junior college education (25.8%). In terms of occupation, enterprise staff accounted for the largest proportion (36.8%), followed by students (14.9%), self-employed or freelance workers (14.1%), and workers or service industry practitioners (14.1%). Monthly income was mainly concentrated in the 5,001–8,000 yuan group (29.8%) and the 8,001–12,000 yuan group (27.9%). In addition, 69.4% of the respondents had lived in Guangzhou for 3 years or longer, suggesting that most participants had sufficient local experience to evaluate the event-related and sport participation context of the 15th National Games (66).

### 4.2 Descriptive statistics, correlations, and common method bias

Descriptive statistics for the main variables are shown in Table 3. The mean values of the six core variables ranged from 3.0226 to 3.5997. Among the psychological constructs, subjective norm had the highest mean value (*M* = 3.5997, SD = 1.10212), followed by perceived behavioral control (*M* = 3.5966, SD = 1.02618), attitude toward sport participation (*M* = 3.5095, SD = 1.07842), and event perception (*M* = 3.4640, SD = 1.08454). Participation intention (*M* = 3.2462, SD = 1.34924) and sport participation behavior (*M* = 3.0226, SD = 1.22536) showed relatively lower mean values. These results indicate that although residents reported moderate to moderately positive event perception and sport-related psychological evaluations, their intention and actual sport participation behavior were comparatively more moderate.

**Table 3:** Descriptive statistics, correlations, and discriminant validity.

Variable	Mean	SD	EP	ATT	SN	PBC	INT	BEH
EP	3.4640	1.08454	0.775					
ATT	3.5095	1.07842	0.468***	0.775				
SN	3.5997	1.10212	0.326***	0.385***	0.825			
PBC	3.5966	1.02618	0.400***	0.318***	0.447***	0.787		
INT	3.2462	1.34924	0.447***	0.461***	0.372***	0.516***	0.819	
BEH	3.0226	1.22536	0.490***	0.493***	0.376***	0.390***	0.497***	0.794

Diagonal values are the square roots of AVE. Lower-triangle values are Pearson correlations. ***p < 0.001. EP, Event perception; ATT, Attitude toward sport participation; SN, Subjective norm; PBC, Perceived behavioral control; INT, Participation intention; BEH, Sport participation behavior.

Because all core variables were collected from the same respondents at a single point in time, common method variance was a potential concern. Several procedural remedies were adopted during questionnaire design and administration. The survey was anonymous, respondents were informed that there were no right or wrong answers, participation was voluntary, and event perception items were separated from sport-participation cognition and behavior items to reduce immediate evaluation consistency. Harman’s single-factor test was then used as a preliminary statistical diagnostic (59). The first unrotated factor explained 36.38% of the total variance, which was below the commonly used threshold of 50%. This result suggests that a single general factor did not dominate the data. However, Harman’s single-factor test cannot fully rule out common method variance; therefore, the findings should be interpreted with caution.

Pearson correlation analysis showed that event perception, attitude toward sport participation, subjective norm, perceived behavioral control, participation intention, and sport participation behavior were all positively and significantly correlated with one another. Event perception was significantly correlated with attitude toward sport participation, subjective norm, perceived behavioral control, participation intention, and sport participation behavior. Participation intention was also significantly correlated with sport participation behavior. These results provided preliminary support for the hypothesized relationships in the extended theory of planned behavior model.

### 4.3 Reliability and validity of the measurement model

The reliability and convergent validity results are presented in Table 4. Cronbach’s alpha coefficients for all constructs exceeded the recommended threshold of 0.70, indicating satisfactory internal consistency (63). Specifically, Cronbach’s alpha was 0.893 for event perception, 0.851 for attitude toward sport participation, 0.843 for subjective norm, 0.855 for perceived behavioral control, 0.866 for participation intention, and 0.861 for sport participation behavior. These results suggest that all measurement scales were reliable.

**Table 4:** Reliability and convergent validity.

Construct	No. of items	Std. factor loading range	Cronbach’s α	CR	AVE
EP	6	0.696–0.889	0.893	0.91	0.60
ATT	5	0.674–0.775	0.851	0.88	0.60
SN	3	0.761–0.828	0.843	0.88	0.68
PBC	4	0.726–0.825	0.855	0.87	0.62
INT	4	0.736–0.842	0.866	0.89	0.67
BEH	4	0.684–0.861	0.861	0.87	0.63

CFA measurement model fit: *χ*^2^/df = 1.698, RMSEA = 0.033, SRMR = 0.058, PNFI = 0.829, CFI = 0.978, IFI = 0.978, TLI = 0.975.

Confirmatory factor analysis was conducted to examine whether the six-factor measurement model fit the observed data (61). The measurement model showed good fit: *χ*^2^/df = 1.698, RMSEA = 0.033, SRMR = 0.058, CFI = 0.978, IFI = 0.978, TLI = 0.975, and PNFI = 0.829. These values met the commonly recommended criteria, indicating that the measurement model provided a satisfactory representation of the data (65).

The standardized factor loadings ranged from 0.674 to 0.889, and all factor loadings were statistically significant. The composite reliability values ranged from 0.87 to 0.91, exceeding the recommended value of 0.70. The average variance extracted values ranged from 0.60 to 0.68, exceeding the recommended value of 0.50. These results indicate satisfactory convergent validity for all constructs (63, 64).

Discriminant validity was assessed using the Fornell–Larcker criterion. As shown in Table 3, the square root of the AVE for each construct was greater than its correlations with other constructs. The square roots of AVE were 0.775 for event perception, 0.775 for attitude toward sport participation, 0.825 for subjective norm, 0.787 for perceived behavioral control, 0.819 for participation intention, and 0.794 for sport participation behavior. Therefore, the constructs were empirically distinct from one another, supporting the discriminant validity of the measurement model. Additional discriminant validity assessment using the heterotrait–monotrait ratio of correlations showed that HTMT values ranged from 0.373 to 0.599. All values were below the conservative threshold of 0.85. Specifically, the HTMT values between event perception and attitude toward sport participation, subjective norm, perceived behavioral control, participation intention, and sport participation behavior were 0.537, 0.375, 0.459, 0.508, and 0.558, respectively. These results provide additional evidence that event perception was empirically distinguishable from the other constructs included in the measurement model. The full HTMT matrix is presented in Supplementary Table S2.

### 4.4 Structural equation model and hypothesis testing

After confirming the reliability and validity of the measurement model, structural equation modeling was conducted to test the hypothesized associations. The structural model fit indices are presented in Table 5. The model showed acceptable fit to the data: *χ*^2^ = 721.511, df = 291, *χ*^2^/df = 2.479, RMSEA = 0.048, SRMR = 0.046, PNFI = 0.826, CFI = 0.952, IFI = 0.946, and TLI = 0.913. These results indicate that the structural model adequately represented the relationships among event perception, attitude toward sport participation, subjective norm, perceived behavioral control, participation intention, and sport participation behavior (65).

**Table 5:** Structural model fit indices.

Fit index	Recommended threshold	Observed value	Conclusion
*χ* ^2^	—	721.511	—
df	—	291	—
*χ*^2^/df	< 3	2.479	Acceptable
RMSEA	< 0.08	0.048	Acceptable
SRMR	< 0.08	0.046	Acceptable
PNFI	> 0.50	0.826	Acceptable
CFI	> 0.90	0.952	Acceptable
IFI	> 0.90	0.946	Acceptable
TLI	> 0.90	0.913	Acceptable

**Figure 2:**
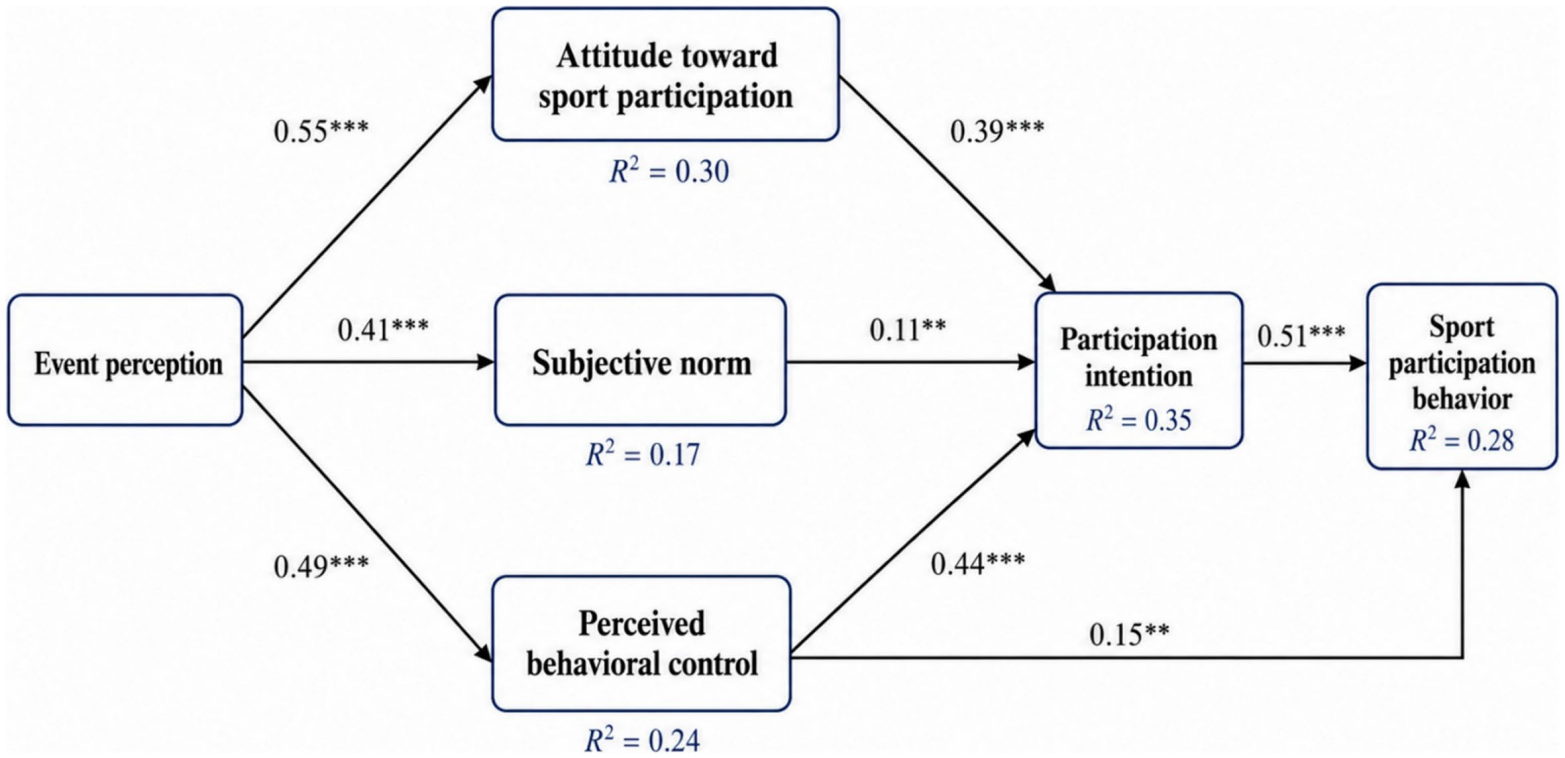
Structural equation model results. Path coefficients indicate standardized associations. ***p < 0.001; **p < 0.01.

The hypothesis testing results are shown in Table 6 and Figure 2. All eight hypothesized associations were statistically significant and in the expected direction. Event perception was positively associated with attitude toward sport participation (*β* = 0.547, *p* < 0.001), subjective norm (*β* = 0.409, *p* < 0.001), and perceived behavioral control (*β* = 0.485, *p* < 0.001), supporting H1, H2, and H3. These results indicate that residents with more favorable perceptions of the 15th National Games were more likely to report positive attitudes toward sport participation, stronger perceived social support, and higher perceived behavioral control.

**Table 6:** Hypothesis testing results.

Hypothesis	Path	Estimate	S. E.	C. R.	*p*	*β*	Result
H1	EP → ATT	0.573	0.051	11.322	***	0.547	Supported
H2	EP → SN	0.429	0.049	8.797	***	0.409	Supported
H3	EP → PBC	0.550	0.052	10.654	***	0.485	Supported
H4	ATT → INT	0.447	0.050	8.957	***	0.390	Supported
H5	SN → INT	0.123	0.044	2.809	0.005	0.107	Supported
H6	PBC → INT	0.462	0.046	10.143	***	0.437	Supported
H7	INT → BEH	0.543	0.057	9.528	***	0.508	Supported
H8	PBC → BEH	0.163	0.056	2.930	0.003	0.145	Supported

*β* represents the standardized path coefficient. All paths are interpreted as associations because the study used cross-sectional data. ***p < 0.001.

Attitude toward sport participation, subjective norm, and perceived behavioral control were all significantly associated with participation intention. Specifically, attitude was positively associated with participation intention (*β* = 0.390, *p* < 0.001), supporting H4. Subjective norm was also positively associated with participation intention (*β* = 0.107, *p* = 0.005), supporting H5. Perceived behavioral control showed the strongest association with participation intention among the three TPB predictors (*β* = 0.437, *p* < 0.001), supporting H6 (67).

Participation intention was positively associated with sport participation behavior (*β* = 0.508, *p* < 0.001), supporting H7. Perceived behavioral control was also directly associated with sport participation behavior (*β* = 0.145, *p* = 0.003), supporting H8. These findings indicate that residents’ self-reported sport participation behavior was associated not only with intention but also with perceived ability and opportunity to engage in regular sport activities (68).

### 4.5 Robustness analysis with demographic covariates

To assess the robustness of the focal associations, five covariate-adjusted hierarchical multiple linear regression models were estimated using the construct scores. Gender, age, educational attainment, monthly income, residence duration, and residential district were entered as demographic covariates in Block 1, followed by the focal theoretical predictor or predictors in Block 2. Categorical demographic variables were dummy coded.

As shown in Table 7, all eight focal associations remained positive and statistically significant after demographic adjustment. Event perception remained positively associated with attitude toward sport participation (adjusted *β* = 0.458, *p* < 0.001), subjective norm (adjusted *β* = 0.295, *p* < 0.001), and perceived behavioral control (adjusted *β* = 0.410, *p* < 0.001). Attitude toward sport participation (adjusted *β* = 0.303, *p* < 0.001), subjective norm (adjusted *β* = 0.086, *p* = 0.023), and perceived behavioral control (adjusted *β* = 0.372, *p* < 0.001) remained positively associated with participation intention. Participation intention (adjusted *β* = 0.387, *p* < 0.001) and perceived behavioral control (adjusted *β* = 0.172, *p* < 0.001) also remained positively associated with sport participation behavior.

**Table 7:** Covariate-adjusted robustness analysis.

Model	Focal association	Adjusted *β*	*t*	*p*	Δ*R*^2^
Model 1	EP → ATT	0.458	12.457	<0.001	0.182
Model 2	EP → SN	0.295	7.431	<0.001	0.075
Model 3	EP → PBC	0.410	10.618	<0.001	0.146
Model 4	ATT → INT	0.303	8.591	<0.001	0.331
Model 4	SN → INT	0.086	2.286	0.023	—
Model 4	PBC → INT	0.372	10.202	<0.001	—
Model 5	INT → BEH	0.387	9.783	<0.001	0.231
Model 5	PBC → BEH	0.172	4.383	<0.001	—

All models were adjusted for gender, age, educational attainment, monthly income, residence duration, and residential district. Categorical demographic variables were dummy coded. Adjusted β represents the standardized regression coefficient. Δ*R*^2^ represents the increase in explained variance after the focal theoretical predictor(s) were entered in Block 2.

The addition of the focal theoretical predictors significantly increased the explained variance across all five models (Δ*R*^2^ = 0.075–0.331; all F-change tests *p* < 0.001). The maximum variance inflation factor across the final models was 3.762. Overall, the direction and statistical significance of the eight focal associations remained substantively consistent with the primary SEM results, indicating that the principal findings were not materially altered after adjustment for the observed demographic characteristics. Full final-model coefficients, including all demographic covariates, are reported in Supplementary Table S3.

### 4.6 Bootstrap analysis of indirect associations

Bootstrap analysis was conducted to examine the hypothesized indirect associations specified in H9a-H9c. The bootstrap procedure used 5,000 resamples and 95% bias-corrected confidence intervals. The results are presented in Table 8. Because the study used a cross-sectional design, these results should be interpreted as indirect associations rather than evidence of causal mediation.

**Table 8:** Bootstrap results for hypothesized indirect associations.

Indirect association path	Indirect effect (β)	S. E.	95% BC CI LL	95% BC CI UL	Result
H9a: EP → ATT → INT → BEH	0.110	0.025	0.064	0.163	Supported
H9b: EP → SN → INT → BEH	0.022	0.009	0.006	0.042	Supported
H9c: EP → PBC → INT → BEH	0.103	0.024	0.061	0.156	Supported

EP, Event perception; ATT, Attitude toward sport participation; SN, Subjective norm; PBC, Perceived behavioral control; INT, Participation intention; BEH, Sport participation behavior. Bootstrap analysis was based on 5,000 resamples and 95% bias-corrected confidence intervals. Indirect associations were considered statistically significant when the confidence interval did not include zero. Because the data were cross-sectional, these results should be interpreted as indirect associations rather than evidence of causal mediation.

Event perception was indirectly associated with sport participation behavior through attitude toward sport participation and participation intention [*β* = 0.110, 95% CI (0.064, 0.163)], supporting H9a. The indirect association through subjective norm and participation intention was also significant [*β* = 0.022, 95% CI (0.006, 0.042)], supporting H9b. In addition, event perception was indirectly associated with sport participation behavior through perceived behavioral control and participation intention [*β* = 0.103, 95% CI (0.061, 0.156)], supporting H9c.

Overall, the bootstrap results suggest that residents with more positive event perception tended to report more favorable sport-related cognition, stronger participation intention, and higher self-reported sport participation behavior. Because the data were cross-sectional, these indirect pathways should be interpreted as statistically significant indirect associations rather than confirmed temporal or causal mechanisms.

## 5 Discussion

### 5.1 Principal findings

This study examined the relationships between event perception and sport participation behavior among host-city residents in Guangzhou by developing an extended TPB model. The findings provide empirical support for the proposed association-based model. Event perception showed positive relationships with attitude toward sport participation, subjective norm, and perceived behavioral control. Attitude, subjective norm, and perceived behavioral control were positively related to participation intention, while participation intention and perceived behavioral control were linked to self-reported sport participation behavior. The bootstrap results further indicated significant indirect associations from event perception to sport participation behavior through TPB constructs and participation intention.

These findings suggest that major sport events may have public-health relevance when residents’ event-related perceptions are linked to sport-related cognition, intention, and perceived feasibility of participation (69). However, the results should not be interpreted as evidence that hosting the 15th National Games caused residents to participate in sport more frequently. The cross-sectional design supports association-based interpretation only. Accordingly, behavioral legacy is discussed in this manuscript as a potential pathway that requires longitudinal verification, not as a confirmed outcome of the event.

### 5.2 Event perception as a contextual antecedent of sport-related cognition

One important finding of this study is that event perception showed significant relationships with the three TPB constructs: attitude toward sport participation, subjective norm, and perceived behavioral control. This supports the positioning of event perception as a contextual antecedent of sport-related cognition (70). However, this should not be interpreted as evidence that the event directly changed residents’ behavior. Rather, residents with more positive perceptions of the 15th National Games tended to report more favorable sport-related cognition.

The positive relationship between event perception and attitude suggests that residents who perceived the Games as visible, meaningful, and valuable in the host-city context were more likely to evaluate sport participation positively. Major sport events may increase the salience of sport in urban life, strengthen sport-related narratives, and connect sport participation with health, community, and city identity. These contextual appraisals may be linked to more favorable evaluations of regular participation.

Event perception also showed a positive relationship with subjective norm. Major sport events are accompanied by media coverage, public discussion, community activities, and interpersonal communication. These processes may increase residents’ perception that sport participation is supported or encouraged by family members, peers, colleagues, and the broader community.

In addition, event perception was positively related to perceived behavioral control. Residents who perceived the Games more positively may have become more aware of sport facilities, community programs, participation opportunities, and the supportive sport atmosphere in Guangzhou. This relationship is particularly relevant because sport participation depends not only on motivation but also on perceived feasibility and access to resources (71).

### 5.3 TPB constructs and participation intention

The results showed that attitude toward sport participation, subjective norm, and perceived behavioral control all showed positive relationships with participation intention. This finding supports the applicability of the TPB for explaining sport participation intention among host-city residents and indicates that intention is related to evaluative, social, and control-related factors (12).

Attitude toward sport participation showed a positive relationship with participation intention. Residents who perceived regular sport participation as beneficial, meaningful, enjoyable, and worthwhile were more likely to report stronger intention to participate. From a public-health perspective, this finding suggests that communication strategies should not only provide factual information about the benefits of physical activity, but also emphasize enjoyment, personal meaning, and everyday relevance (32).

Subjective norm was also related to participation intention, although its coefficient was smaller than those of attitude and perceived behavioral control. This pattern is consistent with many TPB-based studies in which subjective norm is meaningful but not always the strongest predictor. In the context of host-city sport participation, social support from family members, friends, colleagues, peers, and community members may reinforce residents’ intention by making sport participation socially visible and encouraged.

Perceived behavioral control showed a particularly strong relationship with participation intention. This suggests that residents’ intention to participate in sport is closely related to whether they believe participation is feasible in daily life. Barriers such as limited time, work pressure, family responsibilities, lack of nearby facilities, cost, or insufficient confidence may weaken intention even when residents recognize the value of sport participation.

### 5.4 Participation intention, perceived behavioral control, and sport participation behavior

Participation intention showed a positive relationship with sport participation behavior. This finding is broadly consistent with the theoretical relationship between intention and behavior proposed by the TPB. However, the present study did not test prospective behavioral prediction because participation intention referred to the following 4 weeks, whereas sport participation behavior referred to the preceding 4 weeks. Residents who reported stronger plans and willingness to participate in sport also reported higher levels of leisure-time sport and exercise participation. However, this relationship should be interpreted as an association because intention and behavior were measured at the same point in time.

Perceived behavioral control was also directly linked to sport participation behavior. This finding is important because regular sport participation requires not only motivation but also practical feasibility. Time, facility access, physical capacity, financial cost, family responsibilities, and confidence may all affect whether residents can participate regularly (71).

These results suggest that perceived behavioral control may help explain part of the intention-behavior gap in sport participation. Public health strategies related to major sport events should therefore not rely only on motivational campaigns. They should also consider whether residents perceive participation as accessible, affordable, safe, and manageable in their everyday lives (72).

### 5.5 Indirect pathways and the behavioral legacy of major sport events

The bootstrap results showed that event perception was indirectly associated with sport participation behavior through three hypothesized TPB-based routes: an evaluative route through attitude and participation intention, a social route through subjective norm and participation intention, and a control-related route through perceived behavioral control and participation intention. These findings are consistent with the proposed extended TPB model, but they should be interpreted as indirect associations rather than confirmed causal mediation. Because all variables were measured at a single point in time, the analysis cannot establish temporal ordering among event perception, TPB constructs, intention, and behavior.

The evaluative route suggests that residents with more positive event perception tended to report more favorable evaluations of sport participation, stronger intention, and higher self-reported participation. The social route suggests that event-related social atmosphere may be linked to perceived support for sport participation and intention formation. The control-related route suggests that residents who perceived the event context more positively also tended to perceive sport participation as more feasible and manageable.

The control-related route deserves particular attention because perceived behavioral control was linked to both participation intention and sport participation behavior in the structural model. This finding suggests that potential behavioral legacy should not be understood only as inspiration or enthusiasm. It may also depend on enabling conditions, such as perceived access to facilities, affordable programs, flexible time arrangements, and supportive community resources (73). Longitudinal research is needed to determine whether these association-based pathways translate into sustained behavioral change over time (74).

### 5.6 Theoretical implications

This study makes several theoretical contributions. First, it extends research on major sport events by shifting attention from residents’ general event evaluation, support for hosting, and perceived social benefits to the association between event-related appraisal and sport participation outcomes (75). Previous event studies have often emphasized economic, tourism, image, or social impact outcomes. By examining sport participation behavior, this study links major sport event research more directly with a public-health-relevant behavioral domain, while recognizing that the present cross-sectional data cannot establish event-generated behavioral change (69).

Second, this study enriches the TPB by incorporating event perception as a contextual antecedent. The traditional TPB focuses primarily on attitude, subjective norm, perceived behavioral control, intention, and behavior, whereas it pays less attention to the broader social and environmental contexts in which these proximal constructs are formed. By positioning event perception as a contextual appraisal variable, this study explains how a host-city event context may be linked to residents’ sport-related attitudes, perceived social expectations, and perceived behavioral control (70).

Third, this study clarifies the theoretical role of event perception in sport-event legacy research. Event perception is not proposed as a replacement for event inspiration, psychic income, perceived social impact, event involvement, or place attachment. Rather, it is specified as a behaviorally relevant contextual appraisal construct within an extended TPB model. This distinction helps explain why the present study focuses on indirect associations between event perception and self-reported sport and exercise participation rather than treating behavioral legacy as a direct outcome of event hosting.

### 5.7 Practical implications for public health and host-city governance

The practical implications of this study should be interpreted in relation to the observed associations rather than as evidence-tested intervention effects. The findings suggest that event-related public health strategies may be more coherent when they are connected to the psychological constructs examined in the model: attitude, subjective norm, perceived behavioral control, and participation intention.

First, event communication can be designed to strengthen favorable attitudes toward sport participation. Instead of focusing only on competition results, tourism benefits, or city image, host-city communication may highlight the health value, enjoyment, accessibility, and everyday relevance of regular sport participation. Such messages should be linked to concrete opportunities for residents to participate in sport or exercise activities (73).

Second, because subjective norm showed a positive relationship with participation intention, community-level activities may help create a social environment in which sport participation is visible and encouraged. Family-based activities, workplace sport programs, neighborhood exercise groups, school-community collaborations, and peer-led campaigns may increase residents’ perception that sport participation is socially supported and normalized.

Third, perceived behavioral control deserves particular attention because it was linked to both participation intention and sport participation behavior. Host cities should reduce practical barriers by improving access to public sport facilities, extending opening hours, providing affordable or free community programs, offering activities at flexible times, and disseminating clear information about nearby participation opportunities (76). These measures may help residents perceive sport participation as feasible, convenient, and manageable in daily life.

Fourth, event-based promotion should be linked to sustainable community sport services rather than remaining limited to short-term event publicity (77). The present findings suggest that the public-health relevance of major sport events depends not only on enthusiasm or inspiration, but also on whether residents perceive supportive social conditions and enabling environments for regular participation. These practice-oriented implications should be examined more directly in future intervention and longitudinal studies.

### 5.8 Limitations and future research

This study has several limitations. First, the cross-sectional design limits causal inference. Although the proposed model is theoretically grounded, the data cannot establish that event perception preceded changes in attitude, subjective norm, perceived behavioral control, participation intention, or sport participation behavior. The indirect paths identified through bootstrap analysis should therefore be interpreted as indirect associations rather than confirmed temporal mechanisms (45). Because data were collected after the Games, event perception reflected residents’ post-event appraisal and may have been influenced by retrospective evaluation and the recency of the event context. In addition, participation intention referred to the following 4 weeks, whereas sport participation behavior was assessed retrospectively for the preceding 4 weeks. The two measures were therefore not temporally sequenced in a prospective design. Accordingly, the association between participation intention and sport participation behavior should not be interpreted as evidence that the measured intention prospectively predicted the reported behavior. Future studies should use longitudinal designs to examine whether changes in event perception before, during, and after a major sport event relate to subsequent changes in sport-related cognition, intention, and behavior (78, 79).

Second, this study used a non-probability sampling strategy combining convenience and snowball sampling. Although the sample covered all 11 administrative districts of Guangzhou, it may not be fully representative of the broader population of host-city residents. The sample was concentrated among working-age adults and respondents with relatively high educational attainment. Because sport participation, event awareness, health literacy, and access to sport resources may vary across age, education, income, occupation, health status, and facility access, the generalizability of the findings to older residents, less educated groups, lower-income groups, and residents with limited access to sport facilities remains uncertain (76).

Third, sport participation behavior was measured using self-reported ordered items. Although frequency, duration, intensity, and regularity provide useful information about leisure-time sport and exercise participation, self-reported behavior may be affected by recall bias and social desirability bias (80). Future studies could combine self-reported measures with objective indicators, such as wearable device data, facility usage records, mobile health data, or community sport program participation records.

Fourth, common method variance remains a possible limitation because all core variables were collected from the same respondents at the same time. Although procedural remedies were adopted and Harman’s single-factor test did not indicate a dominant single factor, this test alone cannot fully rule out common method variance (81). Future research should consider temporal separation, multi-source data, marker-variable approaches, or longitudinal panel designs.

Fifth, the structural model explained a meaningful but incomplete proportion of variance in the endogenous constructs. This is consistent with the complexity of sport participation as a health-related behavior (82). Future research could incorporate additional determinants, such as previous sport habits, health status, sport identity, place attachment, event involvement, environmental accessibility, neighborhood walkability, work and family constraints, income, and leisure time (83).

Finally, future studies should examine how event perception, sport-related cognition, participation intention, and actual behavior evolve before, during, and after major sport events. Repeated-measure surveys, longitudinal panel designs, and comparisons between host-city and non-host-city residents would provide stronger evidence regarding temporal ordering and possible behavioral change (84, 85). Recent longitudinal research on sport-event residents also illustrates how longitudinal evidence can complement cross-sectional survey findings by strengthening the assessment of temporal relationships (86).

## 6 Conclusion

This study developed and tested an extended Theory of Planned Behavior model to examine the associations between event perception and sport participation behavior among host-city residents of the 15th National Games in Guangzhou. The findings showed that event perception was positively associated with attitude toward sport participation, subjective norm, and perceived behavioral control. These TPB constructs were positively associated with participation intention, while participation intention and perceived behavioral control were associated with self-reported sport participation behavior. The bootstrap results further indicated statistically significant indirect associations between event perception and sport participation behavior through sport-related cognition and participation intention.

The findings suggest that residents’ perceptions of a major sport event may be linked to sport participation intention and self-reported leisure-time sport and exercise participation through TPB constructs. However, because this study used a cross-sectional design, the results should not be interpreted as evidence that the 15th National Games caused behavioral change or generated a confirmed behavioral legacy. Future longitudinal, comparative, and intervention-based research is needed to determine whether and how event-related perceptions can be transformed into sustained sport participation and broader public-health benefits (87).

## Data Availability

The raw data supporting the conclusions of this article will be made available by the authors, without undue reservation.

## References

[1] World Health Organization. WHO Guidelines on Physical Activity and Sedentary Behaviour. Report No.: 978-92-4-001512-8. Geneva: World Health Organization (2020).

[2] World Health Organization. Global action plan on Physical Activity 2018–2030: more Active People for a Healthier World. Report No.: 978-92-4-151418-7. Geneva: World Health Organization (2018).

[3] Wang Z Zhou P Mou C. Advancing universal health coverage through non-medical interventions: the evolution and equity of China's National Fitness policy system. Front Public Health. (2026) 14:1770159. doi: 10.3389/fpubh.2026.1770159, 41799446 PMC12965053

[4] Rhodes RE McEwan D Rebar AL. Theories of physical activity behaviour change: a history and synthesis of approaches. Psychol Sport Exerc. (2019) 42:100–9. doi: 10.1016/j.psychsport.2018.11.010

[5] Fourie J Santana-Gallego M. Mega-sport events and inbound tourism: new data, methods and evidence. Tour Manag Perspect. (2022) 43:101002. doi: 10.1016/j.tmp.2022.101002

[6] Xu H Sato S. Sport events' impact and legacy on quality of life: a scoping review. Front Sports Active Living. (2025) 7:1681463. doi: 10.3389/fspor.2025.1681463, 41245647 PMC12611813

[7] Weed M Coren E Fiore J Wellard I Chatziefstathiou D Dowse S . The Olympic games and raising sport participation: a systematic review of evidence and an interrogation of policy for a demonstration effect. Eur Sport Manag Q. (2015) 15:195–226. doi: 10.1080/16184742.2014.998695

[8] Kaplanidou K Karadakis K Gibson H Thapa B Walker M Geldenhuys S . Quality of life, event impacts, and mega-event support among south African residents before and after the 2010 FIFA world cup. J Travel Res. (2013) 52:631–45. doi: 10.1177/0047287513478501

[9] Schnitzer M Kössler C Schlemmer P Peters M. Influence of event and place image on residents' attitudes toward and support for events. J Hosp Tour Res. (2021) 45:1260–81. doi: 10.1177/1096348020919502

[10] Sato S Kinoshita K Funahashi H Furukawa T Ma S-C Kaplanidou K. A longitudinal study of the impact of the Tokyo 2020 Olympics on Japanese residents' support: the mediating role of social well-being. J Destin Mark Manag. (2024) 34:100947. doi: 10.1016/j.jdmm.2024.100947

[11] Ramchandani G Coleman RJ Bingham J. Sport participation behaviours of spectators attending major sports events and event-induced attitudinal changes towards sport. Int J Event Festiv Manag. (2017) 8:121–35. doi: 10.1108/IJEFM-02-2016-0014

[12] Ajzen I. The theory of planned behavior. Organ Behav Hum Decis Process. (1991) 50:179–211. doi: 10.1016/0749-5978(91)90020-T

[13] Ajzen I. The theory of planned behavior: frequently asked questions. Hum Behav Emerg Technol. (2020) 2:314–24. doi: 10.1002/hbe2.195

[14] Ajzen I Madden TJ. Prediction of goal-directed behavior: attitudes, intentions, and perceived behavioral control. J Exp Soc Psychol. (1986) 22:453–74. doi: 10.1016/0022-1031(86)90045-4

[15] Godin G Kok G. The theory of planned behavior: a review of its applications to health-related behaviors. Am J Health Promot. (1996) 11:87–98. doi: 10.4278/0890-1171-11.2.87, 10163601

[16] Ajzen I. Perceived behavioral control, self-efficacy, locus of control, and the theory of planned behavior. J Appl Soc Psychol. (2002) 32:665–83. doi: 10.1111/j.1559-1816.2002.tb00236.x

[17] Conner M Armitage CJ. Extending the theory of planned behavior: a review and avenues for further research. J Appl Soc Psychol. (1998) 28:1429–64. doi: 10.1111/j.1559-1816.1998.tb01685.x

[18] Jha SS Dobe M Taklikar C Lahiri A. Classroom intervention to improve behavioral intention toward regular physical activity among adolescents. Front Public Health. (2024) 12:1260916. doi: 10.3389/fpubh.2024.1260916, 39171298 PMC11337957

[19] Yang C. Does public spending on sports improve people's health? Empirical evidence from China. Front Public Health. (2026) 14:1744141. doi: 10.3389/fpubh.2026.1744141, 41853165 PMC12992297

[20] Hamilton K Van Dongen A Hagger MS. An extended theory of planned behavior for parent-for-child health behaviors: a meta-analysis. Health Psychol. (2020) 39:863–78. doi: 10.1037/hea0000940, 32597678

[21] McEachan RRC Taylor NJ Harrison R Lawton R Gardner P Conner M. Meta-analysis of the reasoned action approach (RAA) to understanding health behaviors. Ann Behav Med. (2016) 50:592–612. doi: 10.1007/s12160-016-9798-4, 27169555 PMC4933736

[22] Bryman A. Social Research Methods. 5th ed. Oxford: Oxford University Press (2016).

[23] Hagger MS Chatzisarantis NLD Biddle SJH. A meta-analytic review of the theories of reasoned action and planned behavior in physical activity: predictive validity and the contribution of additional variables. J Sport Exerc Psychol. (2002) 24:3–32. doi: 10.1123/jsep.24.1.3

[24] Godin G. The Godin-Shephard leisure-time physical activity questionnaire. Health Fitness J Can. (2011) 4:18–22. doi: 10.14288/hfjc.v4i1.82

[25] St Quinton T Morris B Trafimow D. Untangling the theory of planned behavior's auxiliary assumptions and theoretical assumptions: implications for predictive and intervention studies. New Ideas Psychol. (2021) 60:100818. doi: 10.1016/j.newideapsych.2020.100818

[26] Oshimi D Taks M Agha N. Social impact of events: advancing insights on social impact scales. Eur Sport Manag Q. (2023) 23:1843–62. doi: 10.1080/16184742.2022.2076891

[27] Kim W Jun HM Walker M Drane D. Evaluating the perceived social impacts of hosting large-scale sport tourism events: scale development and validation. Tour Manag. (2015) 48:21–32. doi: 10.1016/j.tourman.2014.10.015

[28] Chen S Xing X Potwarka LR Ramchandani G. Opening the black box of building mass sport and physical activity participation from major sporting events: developing a process model of event inspiration. Eur Sport Manag Q. (2026) 26:117–36. doi: 10.1080/16184742.2025.2518980

[29] Cai J. Can sports events improve residents' psychic income? Front Psychol. (2022) 13:938905. doi: 10.3389/fpsyg.2022.938905, 35928424 PMC9343717

[30] Yamashita R. Mega-Para-sporting event social impacts perceived by Tokyo residents: comparison of residents' vitality. Sustainability. (2021) 13:9311. doi: 10.3390/su13169311

[31] Chalip L Green BC Taks M Misener L. Creating sport participation from sport events: making it happen. Int J Sport Policy Polit. (2017) 9:257–76. doi: 10.1080/19406940.2016.1257496

[32] Phipps DJ Hannan TE Rhodes RE Hamilton K. A dual-process model of affective and instrumental attitudes in predicting physical activity. Psychol Sport Exerc. (2021) 54:101899. doi: 10.1016/j.psychsport.2021.101899

[33] Lee Ludvigsen JA Petersen-Wagner R. From television to YouTube: digitalised sport mega-events in the platform society. Leis Stud. (2023) 42:615–32. doi: 10.1080/02614367.2022.2125557

[34] Scarapicchia TMF Amireault S Faulkner G Sabiston CM. Social support and physical activity participation among healthy adults: a systematic review of prospective studies. Int Rev Sport Exerc Psychol. (2017) 10:50–83. doi: 10.1080/1750984X.2016.1183222

[35] Schulenkorf N Schlenker K. Leveraging sport events to maximize community benefits in low- and middle-income countries. Event Manag. (2017) 21:217–31. doi: 10.3727/152599517X14878772869766

[36] Wang Y Hollett NL. Cognitive, affective, and global attitude toward physical activity with different intensities. Int J Sport Exerc Psychol. (2022) 20:551–68. doi: 10.1080/1612197X.2020.1869803

[37] Pedersen MRL Hansen AF Elmose-Østerlund K. Motives and barriers related to physical activity and sport across social backgrounds: implications for health promotion. Int J Environ Res Public Health. (2021) 18:5810. doi: 10.3390/ijerph18115810, 34071630 PMC8198157

[38] Shen Y Lu C Wang B. The impact of internet use on physical activity among Chinese older adults: the mediating role of social support. Front Public Health. (2025) 13:1492188. doi: 10.3389/fpubh.2025.1492188, 39916702 PMC11801014

[39] Cao Z Yang Y Ding W Huang Z. From physical activity intention to behavior: the moderation role of mental toughness among college students and wage earners. Front Psychol. (2021) 12:584760. doi: 10.3389/fpsyg.2021.584760, 34054630 PMC8160549

[40] Rhodes RE Rebar AL. Conceptualizing and defining the intention construct for future physical activity research. Exerc Sport Sci Rev. (2017) 45:209–16. doi: 10.1249/JES.0000000000000127, 28704222

[41] Gomes AR Gonçalves AM Maddux JE Carneiro L. The intention-behaviour gap: an empirical examination of an integrative perspective to explain exercise behaviour. Int J Sport Exerc Psychol. (2018) 16:607–21. doi: 10.1080/1612197X.2017.1321030

[42] Jones G Norman P. Predicting exercise after university: an application of the reasoned action approach across a significant life transition. Psychol Health Med. (2022) 27:1495–506. doi: 10.1080/13548506.2021.1890160, 33622096

[43] Feil K Fritsch J Rhodes RE. The intention-behaviour gap in physical activity: a systematic review and meta-analysis of the action control framework. Br J Sports Med. (2023) 57:1265–71. doi: 10.1136/bjsports-2022-106640, 37460164

[44] Sheeran P. Intention-behavior relations: a conceptual and empirical review. Eur Rev Soc Psychol. (2002) 12:1–36. doi: 10.1080/14792772143000003

[45] Hayes AF. Introduction to Mediation, Moderation, and Conditional Process Analysis: A Regression-Based Approach. 2nd Edn. New York: Guilford Press (2017).

[46] Teare G Taks M. Sport events for sport participation: a scoping review. Front Sports and ctive Living. (2021) 3:655579. doi: 10.3389/fspor.2021.655579, 34095825 PMC8170037

[47] Francis JJ Eccles MP Johnston M Walker A Grimshaw JM . Constructing Questionnaires Based on the Theory of Planned Behaviour: A Manual for Health Services Researchers. Newcastle upon Tyne: Centre for Health Services Research, University of Newcastle upon Tyne (2004).

[48] Tabachnick BG Fidell LS. Using Multivariate Statistics. 5th ed. Boston, MA: Pearson/Allyn and Bacon (2007).

[49] Biernacki P Waldorf D. Snowball sampling: problems and techniques of chain referral sampling. Sociol Methods Res. (1981) 10:141–63. doi: 10.1177/004912418101000205

[50] Etikan I Musa SA Alkassim RS. Comparison of convenience sampling and purposive sampling. Am J Theor Appl Stat. (2015) 5:1–4. doi: 10.11648/j.ajtas.20160501.11, 42361065

[51] Díaz de Rada V. Internet, phone, mail and mixed-mode surveys: the tailored design method. Reis Rev Esp Investig Sociol. (2016) 154:161–5. doi: 10.1002/9781394260645

[52] Boateng GO Neilands TB Frongillo EA Melgar-Quiñonez HR Young SL. Best practices for developing and validating scales for health, social, and behavioral research: a primer. Front Public Health. (2018) 6:149. doi: 10.3389/fpubh.2018.00149, 29942800 PMC6004510

[53] Kim W Walker M. Measuring the social impacts associated with super bowl XLIII: preliminary development of a psychic income scale. Sport Manag Rev. (2012) 15:91–108. doi: 10.1016/j.smr.2011.05.007

[54] La Barbera F Ajzen I. Instrumental vs. experiential attitudes in the theory of planned behaviour: two studies on intention to perform a recommended amount of physical activity. Int J Sport Exerc Psychol. (2024) 22:632–44. doi: 10.1080/1612197X.2022.2161107

[55] Silsbury Z Goldsmith R Rushton A. Systematic review of the measurement properties of self-report physical activity questionnaires in healthy adult populations. BMJ Open. (2015) 5:e008430. doi: 10.1136/bmjopen-2015-008430, 26373402 PMC4577932

[56] Pallant J. SPSS Survival Manual: A Step by Step Guide to Data Analysis Using IBM SPSS. 7th ed. London: Routledge (2020).

[57] Collier JE. Applied Structural Equation Modeling Using AMOS: Basic to Advanced Techniques. Abingdon: Routledge (2020).

[58] Podsakoff PM MacKenzie SB Lee J-Y Podsakoff NP. Common method biases in behavioral research: a critical review of the literature and recommended remedies. J Appl Psychol. (2003) 88:879–903. doi: 10.1037/0021-9010.88.5.879, 14516251

[59] Harman HH. Modern Factor Analysis. 3rd ed. Chicago, IL: University of Chicago Press (1976).

[60] Cronbach LJ. Coefficient alpha and the internal structure of tests. Psychometrika. (1951) 16:297–334. doi: 10.1007/BF02310555

[61] Anderson JC Gerbing DW. Structural equation modeling in practice: a review and recommended two-step approach. Psychol Bull. (1988) 103:411–23. doi: 10.1037/0033-2909.103.3.411

[62] Hu L-t Bentler PM. Cutoff criteria for fit indexes in covariance structure analysis: conventional criteria versus new alternatives. Struct Equ Model. (1999) 6:1–55. doi: 10.1080/10705519909540118

[63] Hair JF Black WC Babin BJ Anderson RE. Multivariate Data Analysis. 8th ed. Andover: Cengage Learning EMEA (2019).

[64] Fornell C Larcker DF. Evaluating structural equation models with unobservable variables and measurement error. J Mark Res. (1981) 18:39–50. doi: 10.1177/002224378101800104

[65] Kline RB. Principles and Practice of Structural Equation Modeling. 5th ed. New York: Guilford Press (2023).

[66] Johnston M Naylor M Dickson G. Local resident support for hosting a major sport event: the role of perceived personal and community impacts. Eur Sport Manag Q. (2023) 23:877–96. doi: 10.1080/16184742.2021.1937263

[67] Rhodes RE Courneya KS. Threshold assessment of attitude, subjective norm, and perceived behavioral control for predicting exercise intention and behavior. Psychol Sport Exerc. (2005) 6:349–61. doi: 10.1016/j.psychsport.2004.04.002

[68] Hagger MS Cheung MWL Ajzen I Hamilton K. Perceived behavioral control moderating effects in the theory of planned behavior: a meta-analysis. Health Psychol. (2022) 41:155–67. doi: 10.1037/hea0001153, 35143225

[69] Krekel C Kavetsos G Ziebarth NR. Passing on the flame: do mega sports events promote health behaviours? Soc Sci Med. (2025) 377:117921. doi: 10.1016/j.socscimed.2025.117921, 40311500

[70] Schulenkorf N Welty Peachey J Chen G Hergesell A. Event leverage: a systematic literature review and new research agenda. Eur Sport Manag Q. (2024) 24:785–809. doi: 10.1080/16184742.2022.2160477

[71] Jenkin CR Eime RM Westerbeek H van Uffelen JGZ. Sport for adults aged 50+ years: participation benefits and barriers. J Aging Phys Act. (2018) 26:363–71. doi: 10.1123/japa.2017-0092, 28952860

[72] Cui C Yin J. Analysis of the exercise intention-behavior gap among college students using explainable machine learning. Front Public Health. (2025) 13:1613553. doi: 10.3389/fpubh.2025.1613553, 40786156 PMC12331686

[73] Liu Z Zhang S Li L Hu B Liu R Zhao Z . Research on the construction and prediction of China's National Fitness Development Index System under Social Reform. Front Public Health. (2022) 10:878515. doi: 10.3389/fpubh.2022.878515, 35651855 PMC9149160

[74] Karadakis K Kaplanidou K. Legacy perceptions among host and non-host Olympic games residents: a longitudinal study of the 2010 Vancouver Olympic games. Eur Sport Manag Q. (2012) 12:243–64. doi: 10.1080/16184742.2012.680067

[75] Reimers V Chao C-W Magnuson B Kuo A Wang L. Examining the effect of social legacies on support for a formula one sport mega event. Asia Pac J Mark Logist. (2025) 37:3080–96. doi: 10.1108/APJML-09-2024-1366

[76] Fang P Sun L Shi S Ahmed Laar R Lu Y. Influencing factors related to female sports participation under the implementation of Chinese government interventions: an analysis based on the China family panel studies. Front Public Health. (2022) 10:875373. doi: 10.3389/fpubh.2022.875373, 35719610 PMC9201213

[77] Aizawa K Orr M Inoue Y Nagazumi J Yoshida M. Leveraging sport events for sustainable sport participation: how schools contribute to sport development through events. Eur Sport Manag Q. (2023) 23:662–82. doi: 10.1080/16184742.2021.1910326

[78] Amagasa S Kamada M Bauman AE Miyachi M Inoue S. Evaluation of pre-games effects of the Tokyo 2020 Olympic games on Japanese population-level physical activity: a time-series analysis. Int J Behav Nutr Phys Act. (2022) 19:96. doi: 10.1186/s12966-022-01332-x, 35932068 PMC9356482

[79] Kinoshita K Usami S Matsuoka H. Perceived event impacts of the Tokyo 2020 Olympic games on residents' eudaimonic well-being: a longitudinal study of within-person changes and relationships. Sport Manag Rev. (2024) 27:455–79. doi: 10.1080/14413523.2023.2300160

[80] Prince SA Adamo KB Hamel ME Hardt J Connor Gorber S Tremblay M. A comparison of direct versus self-report measures for assessing physical activity in adults: a systematic review. Int J Behav Nutr Phys Act. (2008) 5:56. doi: 10.1186/1479-5868-5-56, 18990237 PMC2588639

[81] Cooper B Eva N Zarea Fazlelahi F Newman A Lee A Obschonka M. Addressing common method variance and endogeneity in vocational behavior research: a review of the literature and suggestions for future research. J Vocat Behav. (2020) 121:103472. doi: 10.1016/j.jvb.2020.103472

[82] Chen K-C Gursoy D Lau KLK. Longitudinal impacts of a recurring sport event on local residents with different level of event involvement. Tour Manag Perspect. (2018) 28:228–38. doi: 10.1016/j.tmp.2018.09.005

[83] Williams LJ Hartman N Cavazotte F. Method variance and marker variables: a review and comprehensive CFA marker technique. Organ Res Methods. (2010) 13:477–514. doi: 10.1177/1094428110366036

[84] Bauman AE Reis RS Sallis JF Wells JC Loos RJF Martin BW. Correlates of physical activity: why are some people physically active and others not? Lancet. (2012) 380:258–71. doi: 10.1016/S0140-6736(12)60735-1, 22818938

[85] Sallis JF Cerin E Conway TL Adams MA Frank LD Pratt M . Physical activity in relation to urban environments in 14 cities worldwide: a cross-sectional study. Lancet. (2016) 387:2207–17. doi: 10.1016/S0140-6736(15)01284-2, 27045735 PMC10833440

[86] Pfitzner R Koenigstorfer J. Quality of life of residents living in a city hosting mega-sport events: a longitudinal study. BMC Public Health. (2016) 16:1102. doi: 10.1186/s12889-016-3777-3, 27769276 PMC5073432

[87] Maxwell SE Cole DA Mitchell MA. Bias in cross-sectional analyses of longitudinal mediation: partial and complete mediation under an autoregressive model. Multivar Behav Res. (2011) 46:816–41. doi: 10.1080/00273171.2011.606716, 26736047

