# Supplementary material for: From spectacle to movement? Associations between major sport event perception and sport participation behavior among Host-City residents
Source: Front Public Health. 2026 Aug 4;14:1904469. doi: 10.3389/fpubh.2026.1904469 (PMC13481720; doi:10.3389/fpubh.2026.1904469)
Supplement: Supplementary file 1 [file Table_1.DOCX]

Supplementary Material

**Supplementary Table S1. Full measurement items and response categories**

| Construct | Item | Measurement item | Response categories / coding |
| --- | --- | --- | --- |
| Event perception | EP1 | The 15th National Games made sport more visible in Guangzhou’s everyday urban life. | 1 = strongly disagree to 5 = strongly agree |
| Event perception | EP2 | The 15th National Games created a clear festive atmosphere and collective excitement around sport. | 1 = strongly disagree to 5 = strongly agree |
| Event perception | EP3 | The 15th National Games made me feel more connected to Guangzhou as a host city. | 1 = strongly disagree to 5 = strongly agree |
| Event perception | EP4 | As a resident of Guangzhou, I felt proud that the 15th National Games was hosted locally. | 1 = strongly disagree to 5 = strongly agree |
| Event perception | EP5 | The 15th National Games improved my positive evaluation of Guangzhou’s city image in the event context. | 1 = strongly disagree to 5 = strongly agree |
| Event perception | EP6 | The 15th National Games enhanced my understanding of sport culture and the public value of sport events. | 1 = strongly disagree to 5 = strongly agree |
| Attitude toward sport participation | ATT1 | For me, maintaining regular sport participation in the next month would be beneficial. | 1 = strongly disagree to 5 = strongly agree |
| Attitude toward sport participation | ATT2 | For me, maintaining regular sport participation in the next month would be worthwhile. | 1 = strongly disagree to 5 = strongly agree |
| Attitude toward sport participation | ATT3 | For me, maintaining regular sport participation in the next month would be pleasant. | 1 = strongly disagree to 5 = strongly agree |
| Attitude toward sport participation | ATT4 | For me, maintaining regular sport participation in the next month would be meaningful. | 1 = strongly disagree to 5 = strongly agree |
| Attitude toward sport participation | ATT5 | For me, maintaining regular sport participation in the next month would be enjoyable. | 1 = strongly disagree to 5 = strongly agree |
| Subjective norm | SN1 | Most people who are important to me support my regular participation in sport in the next month. | 1 = strongly disagree to 5 = strongly agree |
| Subjective norm | SN2 | My family members or friends would approve of my engaging in sport at least three times per week, for at least 30 minutes each time, in the next month. | 1 = strongly disagree to 5 = strongly agree |
| Subjective norm | SN3 | People like me generally maintain regular sport participation in their daily lives. | 1 = strongly disagree to 5 = strongly agree |
| Perceived behavioral control | PBC1 | I am confident that I can maintain regular sport participation in the next month. | 1 = strongly disagree to 5 = strongly agree |
| Perceived behavioral control | PBC2 | Even if I am busy with study, work, or family responsibilities, I can arrange time for sport participation. | 1 = strongly disagree to 5 = strongly agree |
| Perceived behavioral control | PBC3 | Whether I maintain regular sport participation in the next month mainly depends on myself. | 1 = strongly disagree to 5 = strongly agree |
| Perceived behavioral control | PBC4 | For me, engaging in sport at least three times per week, for at least 30 minutes each time, in the next month would be easy to do. | 1 = strongly disagree to 5 = strongly agree |
| Participation intention | INT1 | I intend to engage in sport at least three times per week, for at least 30 minutes each time, in the next four weeks. | 1 = strongly disagree to 5 = strongly agree |
| Participation intention | INT2 | I plan to maintain regular sport participation in the next four weeks. | 1 = strongly disagree to 5 = strongly agree |
| Participation intention | INT3 | I have a clear intention to maintain regular sport participation in the next four weeks. | 1 = strongly disagree to 5 = strongly agree |
| Participation intention | INT4 | I will make an effort to engage in sport at least three times per week, for at least 30 minutes each time, in the next four weeks. | 1 = strongly disagree to 5 = strongly agree |
| Sport participation behavior | BEH1 | In the past four weeks, how many times per week did you participate in sport on average? | 1 = less than once per week; 2 = 1 time per week; 3 = 2 times per week; 4 = 3-4 times per week; 5 = 5 times or more per week |
| Sport participation behavior | BEH2 | In the past four weeks, what was the average duration of each sport participation session? | 1 = less than 10 minutes; 2 = 10-19 minutes; 3 = 20-29 minutes; 4 = 30-59 minutes; 5 = 60 minutes or more |
| Sport participation behavior | BEH3 | In the past four weeks, what was the usual intensity of your sport participation? | 1 = very light; 2 = light; 3 = moderate; 4 = vigorous; 5 = very vigorous |
| Sport participation behavior | BEH4 | In the past four weeks, how many weeks did you meet the standard of “at least three times per week, with each session lasting at least 30 minutes”? | 1 = 0 weeks; 2 = 1 week; 3 = 2 weeks; 4 = 3 weeks; 5 = 4 weeks |

**Supplementary Table S2. Heterotrait–monotrait ratios of correlations among study constructs**

| Construct | EP | ATT | SN | PBC | INT | BEH |
| --- | --- | --- | --- | --- | --- | --- |
| EP | — |  |  |  |  |  |
| ATT | 0.537 | — |  |  |  |  |
| SN | 0.375 | 0.454 | — |  |  |  |
| PBC | 0.459 | 0.373 | 0.528 | — |  |  |
| INT | 0.508 | 0.536 | 0.435 | 0.599 | — |  |
| BEH | 0.558 | 0.576 | 0.439 | 0.455 | 0.575 | — |

**Note.** EP = event perception; ATT = attitude toward sport participation; SN = subjective norm; PBC = perceived behavioral control; INT = participation intention; BEH = sport participation behavior. All HTMT values were below the conservative threshold of 0.85.

**Supplementary Table S3. Covariate-adjusted hierarchical multiple regression robustness analysis.**

**Panel A. Hierarchical model summary**

| Model | DV | Block 1 R² | Final R² | ΔR² | F change | df1 | df2 | p change | Final F | Final model p | Max VIF |
| --- | --- | --- | --- | --- | --- | --- | --- | --- | --- | --- | --- |
| Model 1 | ATT | 0.087 | 0.269 | 0.182 | 155.177 | 1 | 623 | <0.001 | 8.185 | <0.001 | 3.762 |
| Model 2 | SN | 0.075 | 0.150 | 0.075 | 55.223 | 1 | 623 | <0.001 | 3.934 | <0.001 | 3.762 |
| Model 3 | PBC | 0.050 | 0.196 | 0.146 | 112.749 | 1 | 623 | <0.001 | 5.412 | <0.001 | 3.762 |
| Model 4 | INT | 0.068 | 0.399 | 0.331 | 114.028 | 3 | 621 | <0.001 | 13.745 | <0.001 | 3.691 |
| Model 5 | BEH | 0.094 | 0.325 | 0.231 | 106.650 | 2 | 622 | <0.001 | 10.327 | <0.001 | 3.655 |

**Panel B. Full final-model coefficients**

| Model | DV | Predictor | B | SE | Standardized β | t | p | 95% CI for B |
| --- | --- | --- | --- | --- | --- | --- | --- | --- |
| Model 1 | ATT | Constant | 1.941 | 0.210 | — | 9.250 | <0.001 | [1.529, 2.353] |
| Model 1 | ATT | Gender: Female vs Male | 0.193 | 0.080 | 0.089 | 2.410 | 0.016 | [0.036, 0.350] |
| Model 1 | ATT | Age: 18–29 years vs 30–39 years | 0.036 | 0.117 | 0.015 | 0.308 | 0.759 | [-0.194, 0.266] |
| Model 1 | ATT | Age: 40–49 years vs 30–39 years | -0.009 | 0.150 | -0.003 | -0.058 | 0.954 | [-0.304, 0.287] |
| Model 1 | ATT | Age: 50–59 years vs 30–39 years | -0.317 | 0.219 | -0.096 | -1.453 | 0.147 | [-0.747, 0.112] |
| Model 1 | ATT | Age: 60 years and above vs 30–39 years | -0.448 | 0.310 | -0.075 | -1.447 | 0.148 | [-1.057, 0.160] |
| Model 1 | ATT | Education: Junior high school and below vs Bachelor's degree | -0.064 | 0.186 | -0.016 | -0.343 | 0.732 | [-0.429, 0.302] |
| Model 1 | ATT | Education: High school / Technical secondary school vs Bachelor's degree | 0.202 | 0.134 | 0.068 | 1.512 | 0.131 | [-0.060, 0.464] |
| Model 1 | ATT | Education: Junior college vs Bachelor's degree | -0.008 | 0.101 | -0.003 | -0.082 | 0.934 | [-0.207, 0.191] |
| Model 1 | ATT | Education: Postgraduate degree and above vs Bachelor's degree | 0.143 | 0.132 | 0.041 | 1.082 | 0.280 | [-0.116, 0.402] |
| Model 1 | ATT | Income: ≤3000 yuan vs 5001–8000 yuan | 0.141 | 0.141 | 0.050 | 1.003 | 0.316 | [-0.135, 0.418] |
| Model 1 | ATT | Income: 3001–5000 yuan vs 5001–8000 yuan | -0.174 | 0.133 | -0.055 | -1.306 | 0.192 | [-0.436, 0.088] |
| Model 1 | ATT | Income: 8001–12000 yuan vs 5001–8000 yuan | -0.076 | 0.102 | -0.032 | -0.743 | 0.458 | [-0.276, 0.124] |
| Model 1 | ATT | Income: ≥12001 yuan vs 5001–8000 yuan | -0.218 | 0.135 | -0.063 | -1.612 | 0.107 | [-0.483, 0.047] |
| Model 1 | ATT | Residence: 6 months–1 year vs 3–5 years | -0.240 | 0.143 | -0.072 | -1.679 | 0.094 | [-0.521, 0.041] |
| Model 1 | ATT | Residence: 1–3 years vs 3–5 years | -0.123 | 0.121 | -0.045 | -1.020 | 0.308 | [-0.360, 0.114] |
| Model 1 | ATT | Residence: 5–10 years vs 3–5 years | 0.099 | 0.146 | 0.040 | 0.679 | 0.497 | [-0.187, 0.385] |
| Model 1 | ATT | Residence: More than 10 years vs 3–5 years | 0.328 | 0.203 | 0.107 | 1.614 | 0.107 | [-0.071, 0.726] |
| Model 1 | ATT | District: Yuexiu District vs Haizhu District | -0.147 | 0.163 | -0.041 | -0.902 | 0.367 | [-0.466, 0.173] |
| Model 1 | ATT | District: Liwan District vs Haizhu District | -0.054 | 0.158 | -0.015 | -0.340 | 0.734 | [-0.364, 0.257] |
| Model 1 | ATT | District: Tianhe District vs Haizhu District | -0.077 | 0.161 | -0.022 | -0.476 | 0.634 | [-0.394, 0.240] |
| Model 1 | ATT | District: Baiyun District vs Haizhu District | 0.017 | 0.155 | 0.005 | 0.107 | 0.915 | [-0.288, 0.321] |
| Model 1 | ATT | District: Huangpu District vs Haizhu District | -0.328 | 0.167 | -0.087 | -1.965 | 0.050 | [-0.656, -0.000] |
| Model 1 | ATT | District: Panyu District vs Haizhu District | -0.186 | 0.167 | -0.050 | -1.113 | 0.266 | [-0.515, 0.142] |
| Model 1 | ATT | District: Huadu District vs Haizhu District | 0.144 | 0.172 | 0.036 | 0.837 | 0.403 | [-0.193, 0.481] |
| Model 1 | ATT | District: Nansha District vs Haizhu District | 0.055 | 0.177 | 0.013 | 0.309 | 0.757 | [-0.293, 0.402] |
| Model 1 | ATT | District: Conghua District vs Haizhu District | 0.118 | 0.193 | 0.025 | 0.610 | 0.542 | [-0.261, 0.496] |
| Model 1 | ATT | District: Zengcheng District vs Haizhu District | -0.276 | 0.196 | -0.058 | -1.406 | 0.160 | [-0.660, 0.109] |
| Model 1 | ATT | EP | 0.456 | 0.037 | 0.458 | 12.457 | <0.001 | [0.384, 0.527] |
| Model 2 | SN | Constant | 2.573 | 0.231 | — | 11.131 | <0.001 | [2.119, 3.027] |
| Model 2 | SN | Gender: Female vs Male | -0.054 | 0.088 | -0.024 | -0.614 | 0.540 | [-0.227, 0.119] |
| Model 2 | SN | Age: 18–29 years vs 30–39 years | -0.141 | 0.129 | -0.057 | -1.095 | 0.274 | [-0.394, 0.112] |
| Model 2 | SN | Age: 40–49 years vs 30–39 years | -0.149 | 0.166 | -0.056 | -0.901 | 0.368 | [-0.475, 0.176] |
| Model 2 | SN | Age: 50–59 years vs 30–39 years | -0.479 | 0.241 | -0.142 | -1.988 | 0.047 | [-0.952, -0.006] |
| Model 2 | SN | Age: 60 years and above vs 30–39 years | -0.612 | 0.341 | -0.100 | -1.793 | 0.073 | [-1.283, 0.058] |
| Model 2 | SN | Education: Junior high school and below vs Bachelor's degree | 0.230 | 0.205 | 0.056 | 1.120 | 0.263 | [-0.173, 0.632] |
| Model 2 | SN | Education: High school / Technical secondary school vs Bachelor's degree | 0.146 | 0.147 | 0.048 | 0.990 | 0.322 | [-0.143, 0.435] |
| Model 2 | SN | Education: Junior college vs Bachelor's degree | 0.071 | 0.112 | 0.028 | 0.632 | 0.528 | [-0.149, 0.290] |
| Model 2 | SN | Education: Postgraduate degree and above vs Bachelor's degree | -0.026 | 0.145 | -0.007 | -0.176 | 0.860 | [-0.311, 0.260] |
| Model 2 | SN | Income: ≤3000 yuan vs 5001–8000 yuan | 0.365 | 0.155 | 0.127 | 2.353 | 0.019 | [0.060, 0.670] |
| Model 2 | SN | Income: 3001–5000 yuan vs 5001–8000 yuan | -0.030 | 0.147 | -0.009 | -0.202 | 0.840 | [-0.318, 0.259] |
| Model 2 | SN | Income: 8001–12000 yuan vs 5001–8000 yuan | -0.074 | 0.112 | -0.030 | -0.663 | 0.508 | [-0.295, 0.146] |
| Model 2 | SN | Income: ≥12001 yuan vs 5001–8000 yuan | -0.021 | 0.149 | -0.006 | -0.141 | 0.888 | [-0.313, 0.271] |
| Model 2 | SN | Residence: 6 months–1 year vs 3–5 years | -0.113 | 0.158 | -0.033 | -0.715 | 0.475 | [-0.422, 0.197] |
| Model 2 | SN | Residence: 1–3 years vs 3–5 years | -0.036 | 0.133 | -0.013 | -0.274 | 0.784 | [-0.297, 0.224] |
| Model 2 | SN | Residence: 5–10 years vs 3–5 years | 0.286 | 0.161 | 0.112 | 1.777 | 0.076 | [-0.030, 0.601] |
| Model 2 | SN | Residence: More than 10 years vs 3–5 years | 0.371 | 0.224 | 0.119 | 1.658 | 0.098 | [-0.068, 0.810] |
| Model 2 | SN | District: Yuexiu District vs Haizhu District | -0.085 | 0.179 | -0.023 | -0.477 | 0.633 | [-0.437, 0.266] |
| Model 2 | SN | District: Liwan District vs Haizhu District | -0.113 | 0.174 | -0.032 | -0.649 | 0.516 | [-0.455, 0.229] |
| Model 2 | SN | District: Tianhe District vs Haizhu District | -0.112 | 0.178 | -0.031 | -0.629 | 0.529 | [-0.461, 0.237] |
| Model 2 | SN | District: Baiyun District vs Haizhu District | 0.197 | 0.171 | 0.057 | 1.155 | 0.249 | [-0.138, 0.533] |
| Model 2 | SN | District: Huangpu District vs Haizhu District | -0.283 | 0.184 | -0.073 | -1.539 | 0.124 | [-0.644, 0.078] |
| Model 2 | SN | District: Panyu District vs Haizhu District | -0.073 | 0.184 | -0.019 | -0.396 | 0.692 | [-0.435, 0.289] |
| Model 2 | SN | District: Huadu District vs Haizhu District | 0.203 | 0.189 | 0.050 | 1.075 | 0.283 | [-0.168, 0.575] |
| Model 2 | SN | District: Nansha District vs Haizhu District | -0.072 | 0.195 | -0.017 | -0.367 | 0.713 | [-0.455, 0.311] |
| Model 2 | SN | District: Conghua District vs Haizhu District | 0.016 | 0.212 | 0.003 | 0.074 | 0.941 | [-0.401, 0.433] |
| Model 2 | SN | District: Zengcheng District vs Haizhu District | -0.108 | 0.216 | -0.022 | -0.498 | 0.618 | [-0.532, 0.316] |
| Model 2 | SN | EP | 0.299 | 0.040 | 0.295 | 7.431 | <0.001 | [0.220, 0.379] |
| Model 3 | PBC | Constant | 2.269 | 0.209 | — | 10.837 | <0.001 | [1.858, 2.681] |
| Model 3 | PBC | Gender: Female vs Male | 0.164 | 0.080 | 0.079 | 2.050 | 0.041 | [0.007, 0.321] |
| Model 3 | PBC | Age: 18–29 years vs 30–39 years | -0.101 | 0.117 | -0.044 | -0.867 | 0.386 | [-0.331, 0.128] |
| Model 3 | PBC | Age: 40–49 years vs 30–39 years | -0.102 | 0.150 | -0.041 | -0.678 | 0.498 | [-0.396, 0.193] |
| Model 3 | PBC | Age: 50–59 years vs 30–39 years | 0.032 | 0.218 | 0.010 | 0.144 | 0.885 | [-0.397, 0.460] |
| Model 3 | PBC | Age: 60 years and above vs 30–39 years | -0.185 | 0.309 | -0.033 | -0.599 | 0.550 | [-0.793, 0.422] |
| Model 3 | PBC | Education: Junior high school and below vs Bachelor's degree | -0.015 | 0.186 | -0.004 | -0.078 | 0.938 | [-0.379, 0.350] |
| Model 3 | PBC | Education: High school / Technical secondary school vs Bachelor's degree | 0.092 | 0.133 | 0.033 | 0.692 | 0.489 | [-0.169, 0.354] |
| Model 3 | PBC | Education: Junior college vs Bachelor's degree | 0.081 | 0.101 | 0.034 | 0.799 | 0.425 | [-0.118, 0.279] |
| Model 3 | PBC | Education: Postgraduate degree and above vs Bachelor's degree | -0.056 | 0.132 | -0.017 | -0.423 | 0.673 | [-0.314, 0.203] |
| Model 3 | PBC | Income: ≤3000 yuan vs 5001–8000 yuan | 0.194 | 0.140 | 0.072 | 1.381 | 0.168 | [-0.082, 0.470] |
| Model 3 | PBC | Income: 3001–5000 yuan vs 5001–8000 yuan | 0.077 | 0.133 | 0.026 | 0.581 | 0.561 | [-0.184, 0.339] |
| Model 3 | PBC | Income: 8001–12000 yuan vs 5001–8000 yuan | 0.106 | 0.102 | 0.046 | 1.041 | 0.298 | [-0.094, 0.305] |
| Model 3 | PBC | Income: ≥12001 yuan vs 5001–8000 yuan | 0.157 | 0.135 | 0.048 | 1.164 | 0.245 | [-0.108, 0.422] |
| Model 3 | PBC | Residence: 6 months–1 year vs 3–5 years | -0.214 | 0.143 | -0.067 | -1.500 | 0.134 | [-0.495, 0.066] |
| Model 3 | PBC | Residence: 1–3 years vs 3–5 years | -0.202 | 0.120 | -0.077 | -1.677 | 0.094 | [-0.438, 0.035] |
| Model 3 | PBC | Residence: 5–10 years vs 3–5 years | -0.015 | 0.146 | -0.006 | -0.104 | 0.917 | [-0.301, 0.271] |
| Model 3 | PBC | Residence: More than 10 years vs 3–5 years | -0.201 | 0.203 | -0.069 | -0.993 | 0.321 | [-0.599, 0.197] |
| Model 3 | PBC | District: Yuexiu District vs Haizhu District | 0.144 | 0.162 | 0.042 | 0.890 | 0.374 | [-0.174, 0.463] |
| Model 3 | PBC | District: Liwan District vs Haizhu District | -0.074 | 0.158 | -0.022 | -0.471 | 0.638 | [-0.384, 0.235] |
| Model 3 | PBC | District: Tianhe District vs Haizhu District | -0.179 | 0.161 | -0.053 | -1.113 | 0.266 | [-0.496, 0.137] |
| Model 3 | PBC | District: Baiyun District vs Haizhu District | -0.252 | 0.155 | -0.079 | -1.631 | 0.103 | [-0.556, 0.052] |
| Model 3 | PBC | District: Huangpu District vs Haizhu District | -0.003 | 0.167 | -0.001 | -0.018 | 0.986 | [-0.330, 0.324] |
| Model 3 | PBC | District: Panyu District vs Haizhu District | 0.067 | 0.167 | 0.019 | 0.398 | 0.690 | [-0.261, 0.394] |
| Model 3 | PBC | District: Huadu District vs Haizhu District | -0.193 | 0.171 | -0.051 | -1.127 | 0.260 | [-0.530, 0.143] |
| Model 3 | PBC | District: Nansha District vs Haizhu District | -0.219 | 0.177 | -0.055 | -1.237 | 0.217 | [-0.565, 0.128] |
| Model 3 | PBC | District: Conghua District vs Haizhu District | 0.183 | 0.192 | 0.041 | 0.954 | 0.341 | [-0.194, 0.561] |
| Model 3 | PBC | District: Zengcheng District vs Haizhu District | -0.013 | 0.196 | -0.003 | -0.068 | 0.946 | [-0.397, 0.371] |
| Model 3 | PBC | EP | 0.388 | 0.037 | 0.410 | 10.618 | <0.001 | [0.316, 0.459] |
| Model 4 | INT | Constant | 0.029 | 0.269 | — | 0.108 | 0.914 | [-0.499, 0.557] |
| Model 4 | INT | Gender: Female vs Male | -0.074 | 0.091 | -0.027 | -0.822 | 0.412 | [-0.252, 0.103] |
| Model 4 | INT | Age: 18–29 years vs 30–39 years | -0.022 | 0.133 | -0.007 | -0.165 | 0.869 | [-0.283, 0.239] |
| Model 4 | INT | Age: 40–49 years vs 30–39 years | -0.248 | 0.171 | -0.077 | -1.450 | 0.148 | [-0.583, 0.088] |
| Model 4 | INT | Age: 50–59 years vs 30–39 years | -0.523 | 0.247 | -0.127 | -2.120 | 0.034 | [-1.007, -0.039] |
| Model 4 | INT | Age: 60 years and above vs 30–39 years | -0.083 | 0.352 | -0.011 | -0.235 | 0.814 | [-0.773, 0.608] |
| Model 4 | INT | Education: Junior high school and below vs Bachelor's degree | -0.194 | 0.212 | -0.039 | -0.917 | 0.359 | [-0.610, 0.222] |
| Model 4 | INT | Education: High school / Technical secondary school vs Bachelor's degree | 0.143 | 0.152 | 0.039 | 0.945 | 0.345 | [-0.155, 0.442] |
| Model 4 | INT | Education: Junior college vs Bachelor's degree | -0.052 | 0.115 | -0.017 | -0.453 | 0.651 | [-0.278, 0.174] |
| Model 4 | INT | Education: Postgraduate degree and above vs Bachelor's degree | -0.165 | 0.150 | -0.038 | -1.104 | 0.270 | [-0.459, 0.129] |
| Model 4 | INT | Income: ≤3000 yuan vs 5001–8000 yuan | -0.136 | 0.161 | -0.039 | -0.849 | 0.396 | [-0.452, 0.179] |
| Model 4 | INT | Income: 3001–5000 yuan vs 5001–8000 yuan | -0.135 | 0.151 | -0.034 | -0.893 | 0.372 | [-0.432, 0.162] |
| Model 4 | INT | Income: 8001–12000 yuan vs 5001–8000 yuan | 0.168 | 0.116 | 0.056 | 1.451 | 0.147 | [-0.059, 0.395] |
| Model 4 | INT | Income: ≥12001 yuan vs 5001–8000 yuan | 0.124 | 0.154 | 0.029 | 0.807 | 0.420 | [-0.178, 0.426] |
| Model 4 | INT | Residence: 6 months–1 year vs 3–5 years | -0.036 | 0.163 | -0.009 | -0.219 | 0.826 | [-0.356, 0.284] |
| Model 4 | INT | Residence: 1–3 years vs 3–5 years | -0.125 | 0.137 | -0.036 | -0.912 | 0.362 | [-0.395, 0.144] |
| Model 4 | INT | Residence: 5–10 years vs 3–5 years | -0.085 | 0.166 | -0.027 | -0.513 | 0.608 | [-0.411, 0.241] |
| Model 4 | INT | Residence: More than 10 years vs 3–5 years | 0.108 | 0.228 | 0.028 | 0.471 | 0.638 | [-0.341, 0.556] |
| Model 4 | INT | District: Yuexiu District vs Haizhu District | -0.267 | 0.185 | -0.060 | -1.445 | 0.149 | [-0.629, 0.096] |
| Model 4 | INT | District: Liwan District vs Haizhu District | -0.129 | 0.180 | -0.030 | -0.720 | 0.472 | [-0.482, 0.223] |
| Model 4 | INT | District: Tianhe District vs Haizhu District | 0.055 | 0.184 | 0.013 | 0.301 | 0.764 | [-0.305, 0.416] |
| Model 4 | INT | District: Baiyun District vs Haizhu District | -0.243 | 0.177 | -0.058 | -1.373 | 0.170 | [-0.591, 0.105] |
| Model 4 | INT | District: Huangpu District vs Haizhu District | 0.045 | 0.190 | 0.009 | 0.234 | 0.815 | [-0.329, 0.419] |
| Model 4 | INT | District: Panyu District vs Haizhu District | 0.081 | 0.190 | 0.017 | 0.425 | 0.671 | [-0.293, 0.455] |
| Model 4 | INT | District: Huadu District vs Haizhu District | 0.213 | 0.196 | 0.043 | 1.090 | 0.276 | [-0.171, 0.598] |
| Model 4 | INT | District: Nansha District vs Haizhu District | -0.124 | 0.201 | -0.024 | -0.615 | 0.539 | [-0.519, 0.272] |
| Model 4 | INT | District: Conghua District vs Haizhu District | -0.185 | 0.219 | -0.031 | -0.845 | 0.398 | [-0.615, 0.245] |
| Model 4 | INT | District: Zengcheng District vs Haizhu District | 0.079 | 0.223 | 0.013 | 0.354 | 0.724 | [-0.359, 0.516] |
| Model 4 | INT | ATT | 0.380 | 0.044 | 0.303 | 8.591 | <0.001 | [0.293, 0.466] |
| Model 4 | INT | SN | 0.105 | 0.046 | 0.086 | 2.286 | 0.023 | [0.015, 0.195] |
| Model 4 | INT | PBC | 0.489 | 0.048 | 0.372 | 10.202 | <0.001 | [0.395, 0.583] |
| Model 5 | BEH | Constant | 1.283 | 0.235 | — | 5.454 | <0.001 | [0.821, 1.745] |
| Model 5 | BEH | Gender: Female vs Male | -0.188 | 0.087 | -0.076 | -2.163 | 0.031 | [-0.358, -0.017] |
| Model 5 | BEH | Age: 18–29 years vs 30–39 years | -0.050 | 0.128 | -0.018 | -0.389 | 0.698 | [-0.301, 0.201] |
| Model 5 | BEH | Age: 40–49 years vs 30–39 years | -0.034 | 0.165 | -0.011 | -0.205 | 0.838 | [-0.357, 0.289] |
| Model 5 | BEH | Age: 50–59 years vs 30–39 years | -0.318 | 0.236 | -0.085 | -1.344 | 0.179 | [-0.782, 0.146] |
| Model 5 | BEH | Age: 60 years and above vs 30–39 years | -0.674 | 0.336 | -0.099 | -2.005 | 0.045 | [-1.334, -0.014] |
| Model 5 | BEH | Education: Junior high school and below vs Bachelor's degree | -0.027 | 0.203 | -0.006 | -0.135 | 0.893 | [-0.427, 0.372] |
| Model 5 | BEH | Education: High school / Technical secondary school vs Bachelor's degree | 0.000 | 0.146 | 0.000 | 0.001 | 0.999 | [-0.287, 0.287] |
| Model 5 | BEH | Education: Junior college vs Bachelor's degree | 0.026 | 0.111 | 0.009 | 0.238 | 0.812 | [-0.191, 0.244] |
| Model 5 | BEH | Education: Postgraduate degree and above vs Bachelor's degree | -0.049 | 0.144 | -0.013 | -0.342 | 0.733 | [-0.332, 0.233] |
| Model 5 | BEH | Income: ≤3000 yuan vs 5001–8000 yuan | 0.390 | 0.154 | 0.122 | 2.534 | 0.012 | [0.088, 0.692] |
| Model 5 | BEH | Income: 3001–5000 yuan vs 5001–8000 yuan | 0.035 | 0.145 | 0.010 | 0.239 | 0.812 | [-0.251, 0.320] |
| Model 5 | BEH | Income: 8001–12000 yuan vs 5001–8000 yuan | 0.010 | 0.111 | 0.004 | 0.093 | 0.926 | [-0.208, 0.229] |
| Model 5 | BEH | Income: ≥12001 yuan vs 5001–8000 yuan | -0.131 | 0.148 | -0.033 | -0.887 | 0.375 | [-0.421, 0.159] |
| Model 5 | BEH | Residence: 6 months–1 year vs 3–5 years | -0.326 | 0.157 | -0.086 | -2.083 | 0.038 | [-0.634, -0.019] |
| Model 5 | BEH | Residence: 1–3 years vs 3–5 years | -0.295 | 0.132 | -0.094 | -2.231 | 0.026 | [-0.554, -0.035] |
| Model 5 | BEH | Residence: 5–10 years vs 3–5 years | 0.052 | 0.159 | 0.018 | 0.325 | 0.746 | [-0.261, 0.364] |
| Model 5 | BEH | Residence: More than 10 years vs 3–5 years | 0.388 | 0.217 | 0.112 | 1.788 | 0.074 | [-0.038, 0.814] |
| Model 5 | BEH | District: Yuexiu District vs Haizhu District | 0.132 | 0.177 | 0.033 | 0.746 | 0.456 | [-0.216, 0.481] |
| Model 5 | BEH | District: Liwan District vs Haizhu District | 0.125 | 0.173 | 0.032 | 0.726 | 0.468 | [-0.214, 0.465] |
| Model 5 | BEH | District: Tianhe District vs Haizhu District | -0.162 | 0.177 | -0.041 | -0.919 | 0.358 | [-0.509, 0.184] |
| Model 5 | BEH | District: Baiyun District vs Haizhu District | -0.114 | 0.170 | -0.030 | -0.668 | 0.504 | [-0.447, 0.220] |
| Model 5 | BEH | District: Huangpu District vs Haizhu District | -0.171 | 0.182 | -0.040 | -0.938 | 0.349 | [-0.529, 0.187] |
| Model 5 | BEH | District: Panyu District vs Haizhu District | -0.237 | 0.183 | -0.055 | -1.295 | 0.196 | [-0.596, 0.122] |
| Model 5 | BEH | District: Huadu District vs Haizhu District | 0.074 | 0.188 | 0.017 | 0.396 | 0.693 | [-0.295, 0.444] |
| Model 5 | BEH | District: Nansha District vs Haizhu District | 0.177 | 0.194 | 0.037 | 0.913 | 0.362 | [-0.204, 0.557] |
| Model 5 | BEH | District: Conghua District vs Haizhu District | -0.114 | 0.211 | -0.021 | -0.542 | 0.588 | [-0.527, 0.299] |
| Model 5 | BEH | District: Zengcheng District vs Haizhu District | 0.265 | 0.214 | 0.049 | 1.236 | 0.217 | [-0.156, 0.685] |
| Model 5 | BEH | INT | 0.352 | 0.036 | 0.387 | 9.783 | <0.001 | [0.281, 0.422] |
| Model 5 | BEH | PBC | 0.205 | 0.047 | 0.172 | 4.383 | <0.001 | [0.113, 0.297] |

**Note.** N = 652. The primary SEM did not include demographic covariates. For the robustness analysis, gender, age, educational attainment, monthly income, residence duration, and residential district were entered in Block 1, and the focal theoretical predictor(s) were entered in Block 2. Categorical demographic variables were dummy coded. Reference categories were male, 30–39 years, bachelor's degree, monthly income of 5,001–8,000 yuan, residence duration of 3–5 years, and Haizhu District. B = unstandardized regression coefficient; SE = standard error; β = standardized regression coefficient; CI = confidence interval; DV = dependent variable; EP = event perception; ATT = attitude toward sport participation; SN = subjective norm; PBC = perceived behavioral control; INT = participation intention; BEH = sport participation behavior.
